# Global, regional, and national burden of brain and other CNS cancer, 1990–2016: a systematic analysis for the Global Burden of Disease Study 2016

**DOI:** 10.1016/S1474-4422(18)30468-X

**Published:** 2019-04

**Authors:** GBD 2016 Brain and Other CNS Cancer Collaborators, Anoop P Patel, James L Fisher, Emma Nichols, Foad Abd-Allah, Jemal Abdela, Ahmed Abdelalim, Haftom Niguse Abraha, Dominic Agius, Fares Alahdab, Tahiya Alam, Christine A Allen, Nahla Hamed Anber, Ashish Awasthi, Hamid Badali, Abate Bekele Belachew, Ali Bijani, Tone Bjørge, Félix Carvalho, Ferrán Catalá-López, Jee-Young J Choi, Ahmad Daryani, Meaza Girma Degefa, Gebre Teklemariam Demoz, Huyen Phuc Do, Manisha Dubey, Eduarda Fernandes, Irina Filip, Kyle J Foreman, Abadi Kahsu Gebre, Yilma Chisha Dea Geramo, Nima Hafezi-Nejad, Samer Hamidi, James D Harvey, Hamid Yimam Hassen, Simon I Hay, Seyed Sina Naghibi Irvani, Mihajlo Jakovljevic, Ravi Prakash Jha, Amir Kasaeian, Ibrahim A Khalil, Ejaz Ahmad Khan, Young-Ho Khang, Yun Jin Kim, Getnet Mengistu, Karzan Abdulmuhsin Mohammad, Ali H Mokdad, Gabriele Nagel, Mohsen Naghavi, Gurudatta Naik, Huong Lan Thi Nguyen, Long Hoang Nguyen, Trang Huyen Nguyen, Molly R Nixon, Andrew T Olagunju, David M Pereira, Gabriel David Pinilla-Monsalve, Hossein Poustchi, Mostafa Qorbani, Amir Radfar, Robert C Reiner, Gholamreza Roshandel, Hosein Safari, Saeid Safiri, Abdallah M Samy, Shahabeddin Sarvi, Masood Ali Shaikh, Mehdi Sharif, Rajesh Sharma, Sara Sheikhbahaei, Reza Shirkoohi, Jasvinder A Singh, Mari Smith, Rafael Tabarés-Seisdedos, Bach Xuan Tran, Khanh Bao Tran, Irfan Ullah, Elisabete Weiderpass, Kidu Gidey Weldegwergs, Ebrahim M Yimer, Vesna Zadnik, Zoubida Zaidi, Richard G Ellenbogen, Theo Vos, Valery L Feigin, Christopher J L Murray, Christina Fitzmaurice

## Abstract

**Background:**

Brain and CNS cancers (collectively referred to as CNS cancers) are a source of mortality and morbidity for which diagnosis and treatment require extensive resource allocation and sophisticated diagnostic and therapeutic technology. Previous epidemiological studies are limited to specific geographical regions or time periods, making them difficult to compare on a global scale. In this analysis, we aimed to provide a comparable and comprehensive estimation of the global burden of brain cancer between 1990 and 2016.

**Methods:**

We report means and 95% uncertainty intervals (UIs) for incidence, mortality, and disability-adjusted life-years (DALYs) estimates for CNS cancers (according to the International Classification of Diseases tenth revision: malignant neoplasm of meninges, malignant neoplasm of brain, and malignant neoplasm of spinal cord, cranial nerves, and other parts of CNS) from the Global Burden of Diseases, Injuries, and Risk Factors Study 2016. Data sources include vital registration and cancer registry data. Mortality was modelled using an ensemble model approach. Incidence was estimated by dividing the final mortality estimates by mortality to incidence ratios. DALYs were estimated by summing years of life lost and years lived with disability. Locations were grouped into quintiles based on the Socio-demographic Index (SDI), a summary indicator of income per capita, years of schooling, and total fertility rate.

**Findings:**

In 2016, there were 330 000 (95% UI 299 000 to 349 000) incident cases of CNS cancer and 227 000 (205 000 to 241 000) deaths globally, and age-standardised incidence rates of CNS cancer increased globally by 17·3% (95% UI 11·4 to 26·9) between 1990 and 2016 (2016 age-standardised incidence rate 4·63 per 100 000 person-years [4·17 to 4·90]). The highest age-standardised incidence rate was in the highest quintile of SDI (6·91 [5·71 to 7·53]). Age-standardised incidence rates increased with each SDI quintile. East Asia was the region with the most incident cases of CNS cancer for both sexes in 2016 (108 000 [95% UI 98 000 to 122 000]), followed by western Europe (49 000 [37 000 to 54 000]), and south Asia (31 000 [29 000 to 37 000]). The top three countries with the highest number of incident cases were China, the USA, and India. CNS cancer was responsible for 7·7 million (95% UI 6·9 to 8·3) DALYs globally, a non-significant change in age-standardised DALY rate of −10·0% (−16·4 to 2·6) between 1990 and 2016. The age-standardised DALY rate decreased in the high SDI quintile (−10·0% [–27·1 to −0·1]) and high-middle SDI quintile (−10·5% [–18·4 to −1·4]) over time but increased in the low SDI quintile (22·5% [11·2 to 50·5]).

**Interpretation:**

CNS cancer is responsible for substantial morbidity and mortality worldwide, and incidence increased between 1990 and 2016. Significant geographical and regional variation in the incidence of CNS cancer might be reflective of differences in diagnoses and reporting practices or unknown environmental and genetic risk factors. Future efforts are needed to analyse CNS cancer burden by subtype.

**Funding:**

Bill & Melinda Gates Foundation.

## Introduction

Primary brain and CNS cancers (collectively called CNS cancer from this point onwards) affect both children and adults and are diagnosed in all anatomical regions of the CNS, with the vast majority (>90%) occurring in the brain and the remainder occurring in the meninges, spinal cord, and cranial nerves. They represent a substantial source of morbidity and mortality worldwide.^1^ The effect that CNS cancer has on health-care systems is out of proportion with incidence due to the high rates of mortality and inherently disabling effects it has on patients, often preventing independent functioning.^2^ The signs and symptoms associated with CNS cancer are heterogeneous, dependent on histopathology and affected anatomical regions, and include headaches, vision loss, seizures, speech disturbance, and paralysis. The burden of CNS cancer is compounded by the fact that effective treatment is multimodal and requires access to neurosurgical care, radiation, and chemotherapy. This highly specialised care for CNS cancer is not widely available in many areas of the world.^3^, ^4^ In these contexts, it is important to understand the distribution of CNS cancer to inform local, national, and international efforts to allocate health-care resources appropriately.

**Research in context**

**Evidence before this study**

Global Burden of Diseases, Injuries, and Risk Factors (GBD) Study 2016 is an update of the GBD 2015 study. GBD 2015 provided estimates on 249 causes of death for 195 countries and territories from 1980 to 2015. For GBD 2016 we added 6748 site years of data sources (from 12 220 site years to 18 968 site years) that were used in the brain and CNS cancer (collectively called CNS cancer from this point onwards) estimation process. For the GBD cancer estimation, we comprehensively searched for vital registration system and cancer registry data rather than using published literature. The International Agency for Research on Cancer produces periodically updated estimates for all cancers including CNS cancers in the GLOBOCAN project. GLOBOCAN does not include estimates over time and does not include estimates for years of life lost, years lived with disability, or disability-adjusted life-years (DALYs).

**Added value of this study**

The GBD 2016 study reports disease burden for more than 300 diseases and injuries across 195 countries from 1990 to 2016. In this Article, we report the detailed results for CNS cancer incidence, mortality, and DALYs by age, sex, and Socio-demographic Index (SDI) from the GBD 2016 study.

**Implications of all the available evidence**

Between 1990 and 2016, the number of incident cases of CNS cancer have increased across all geographical regions and SDI quintiles, except for eastern Europe where incident cases have remained stable. However, probably because of access to early detection and care, the mortality to incidence ratio decreases with improvements in SDI. Reasons for the large heterogeneity in incidence remain unclear and need further investigation. This study provides quantitative estimates on distribution of disease burden related to CNS cancer across the globe and can inform resource allocation and cancer control strategies.

The most common histological type of primary CNS cancer is glioma—a group of malignant brain tumours, including high-grade glioma or glioblastoma and low-grade gliomas (astrocytoma, oligodendroglioma). The remainder is made up of various histologies, including other tumours of glial origin (ependymomas, schwannomas), medulloblastomas, CNS lymphomas, and meningiomas.^5^ Glioblastomas, the most common primary brain cancer of glial origin, are almost universally fatal within 2 years of diagnosis despite maximal surgical and medical therapy.^6^ In children, the most common histologies include astrocytoma, medulloblastoma, germ-cell tumours, brainstem gliomas, and ependymomas. Although these diseases are associated with substantial morbidity, long-term survival is possible with comprehensive treatment strategies.^7^

Few known risk factors are associated with CNS cancer. The only consistent associations that have resulted from epidemiological studies are positive associations with ionising radiation (atomic weapon radiation, previous therapeutic irradiation) and negative associations with atopic conditions (asthma, eczema, food allergies).^8^, ^9^ A multitude of other risk factors have been considered, including cell phone radiation, aspirin use, hormonal factors, low-frequency magnetic fields, pesticides, dietary factors, and industrial exposures, none of which have consistently shown associations with risk of CNS cancer.^10^, ^11^ Various genetic syndromes and associated low-frequency alleles are associated with increased risk of CNS cancer, but these account for only a minute fraction of the total cases.^12^, ^13^ Increased understanding of the distribution of CNS cancer across geographical regions might shed light on extrinsic factors and their contribution to the epidemiological pattern.

Previous efforts to quantify the incidence of CNS cancer are limited to specific countries, geographical regions, or single-year estimates.^14^ A meta-analysis^15^ of available studies concluded that more standardised approaches to study the epidemiology of these tumours is needed. The Global Burden of Diseases, Injuries, and Risk Factors (GBD) study aims to quantify health loss due to all diseases from 1990 to the most recent year with annual updates. In this analysis, we used data from the GBD 2016 Study to quantify the incidence, mortality, and disability-adjusted life-years (DALYs) of CNS cancer across the world by sex and 5-year age group, to explore potential relationships with development status using the Socio-demographic Index (SDI), which is a composite indicator of fertility, income, and education.

## Methods

### Overview

Methods to estimate CNS cancer incidence, mortality, prevalence, years lived with disability (YLDs), years of life lost (YLLs), and DALYs have been previously described in detail.^16^, ^17^, ^18^, ^19^ Additional information is included in the appendix 1. In GBD 2016, CNS cancer includes all cancers coded as C70.0–C72.9 (C70, malignant neoplasm of meninges; C71, malignant neoplasm of brain; C72, malignant neoplasm of spinal cord, cranial nerves, and other parts of the CNS) in the International Classification of Diseases (ICD) tenth revision. Since the ICD is based on the site of the cancer rather than histology, the GBD study currently does not include any estimates for brain and CNS cancer subtypes. All rates are reported per 100 000 person-years. All estimates were produced from 1990 to 2016 and are reported with 95% uncertainty intervals (UIs), which were derived from the 2·5th and 97·5th percentile of 1000 draws. Results were considered significantly different if confidence intervals did not overlap. The study was compliant with GATHER guidelines.^20^

### Mortality and YLLs estimation

In summary, the methodological framework starts with estimating CNS cancer mortality. We used any source that provided a representative partial or complete sample of incidence or mortality data. Data sources included vital registration systems cancer registry and verbal autopsy data. We mapped different coding systems to the GBD cause list. Data were provided by collaborators or accessed via publicly available sources. Since mortality data can be sparse, and incidence data from registries often exist in locations without mortality registration, we transformed incidence data to mortality estimates by multiplying the registry incidence data with the corresponding, independently modelled, mortality to incidence ratio.^21^ We modelled mortality to incidence ratios using input data from locations where CNS cancer mortality and incidence data were reported for the same year. The initial mortality to incidence ratio model used a linear-step mixed-effects model with a logit link function and the SDI as the predictive covariate. We then smoothed predictions from the linear step over space and time and adjusted in a Gaussian process regression. We used the combined data (observed vital registration deaths and deaths derived from registry incidence multiplied by mortality to incidence ratio) on CNS cancer mortality as data inputs for a cause of death ensemble model approach (CODEm).^22^ The covariates we used in the model, with an assumption built in that these predictors have a positive association with deaths from CNS cancer, included alcohol (L per capita), cigarette use (cumulative cigarette use and smoking prevalence), red meat and saturated fat consumption, mean total cholesterol per capita, and systolic blood pressure. We used lagged distributed income as a covariate without a previous assumption on the direction of the relationship. We used SDI, fruit and vegetable consumption, education, and the Healthcare Access and Quality index^23^ in the model with a negative prior (reverse correlation). Of note, covariates used in the CODEm modelling process do not need to have a proven causal relationship, but there must be a plausible relationship between the covariates and CNS cancer death.^22^ CODEm is designed to choose among the predictors that produce the best fit to the input data. YLLs were estimated as the multiplication of counts of death and a standard, ideal, remaining life expectancy at the age of death.^16^

### Incidence, prevalence, and YLDs estimation

We estimated CNS cancer incidence by dividing the final mortality estimates by the mortality to incidence ratio. We derived 10-year CNS cancer prevalence by estimating survival for each incidence cohort. We modelled survival using the mortality to incidence ratio as a scalar to determine where countries were placed between a theoretical best-case and worst-case survival. We estimated prevalence from incidence rather than using prevalence data in the estimation process due to the scarcity of prevalence data for most countries. We considered the prevalence cohort beyond 10 years as cured. We then divided the prevalence of the cured population into two phases (diagnosis and primary therapy, and controlled or remission phase). We divided the prevalence for the cohort that died within 10 years into four phases (diagnosis and primary therapy, controlled phase, disseminated or metastatic, and terminal phase). We used a fixed duration of 5 months for the diagnosis and primary therapy phase, 7 months for the disseminated or metastatic phase, and 1 month for the terminal phase. We assigned the remaining prevalence time to the controlled phase. For each phase, we multiplied prevalence with a distinct disability weight to estimate YLDs.^24^ Disability weights range from 0–1 and reflect the relative severity of time lived by a person in a health state compared with all other health states quantified in GBD. A disability weight of 0·29 (95% UI 0·19–0·40) was used for diagnosis and primary therapy, 0·05 (0·03–0·07) for controlled phase, 0·45 (0·31–0·60) for disseminated or metastatic phase, and 0·54 (0·38–0·69) for terminal phase.

### DALY estimation and effect of SDI

We estimated DALYs by summing YLDs and YLLs by age, sex, location, and year. To examine the effect of the SDI on survival, we analysed the association between the age-standardised mortality to incidence ratio (a surrogate for survival) and SDI for GBD regions.^25^

### Role of the funding source

The funder of the study had no role in study design, data collection, data analysis, data interpretation, or the writing of the report. All authors had full access to the data in the study and had final responsibility for the decision to submit for publication.

## Results

All GBD CNS cancer estimates (incidence, mortality, prevalence, YLLs, YLDs, DALYs) for 1980 through 2016 are available online from GBD Compare and GBD Results Tool, and appendix 2.

In 2016 at the global level, there were 330 000 (95% UI 299 000 to 349 000) incident cases of CNS cancer, with an age-standardised incidence rate of 4·63 per 100 000 person-years (95% UI 4·17 to 4·90), which significantly increased by 17·3% (95% UI 11·4 to 26·9) between 1990 and 2016. CNS cancer was responsible for 227 000 (205 000 to 241 000) deaths globally with an age-standardised death rate of 3·24 per 100 000 person-years (2·91 to 3·43), which did not change significantly between 1990 and 2016 (2·2% (−7·7 to 8·0). CNS cancer was responsible for 7·7 million (6·9 to 8·3) DALYs at the global level, with an age-standardised rate of 105·05 DALYs per 100 000 person-years (94·86 to 113·35; table; appendix 2). The age-standardised DALY rate between 1990 and 2016 decreased by 10·0%, which was not significant (−16·4 to 2·6; table).

**Table:** Deaths, incident cases, and DALYs for CNS cancer in 2016 and percentage change between 1990 and 2016 in age-standardised rates by location

	**Deaths (95% UI)**		**Incidence (95% UI)**		**DALYs (95% UI)**	
	2016 counts	Percentage change in age-standardised rates between 1990 and 2016	2016 counts	Percentage change in age-standardised rates between 1990 and 2016	2016 counts	Percentage change in age-standardised rates between 1990 and 2016
**Global**	**227 039 (204 784 to 241 279)**	**−2·2 (−7·7 to 8·0)**	**329 673 (298 926 to 348 845)**	**17·3 (11·4 to 26·9)**	**7 659 974 (6 922 776 to 8 280 367)**	**−10·0 (−16·4 to 2·6)**
Low SDI	9972 (8653 to 11 208)	26·3 (10·5 to 63·4)	9749 (8616 to 10 810)	9·3 (1·2 to 35·6)	448 065 (390 748 to 505 097)	22·5 (11·2 to 50·5)
Low-middle SDI	36 142 (32 641 to 41 465)	15·4 (0·0 to 74·7)	41 107 (37 077 to 46 399)	7·0 (−5·7 to 61·4)	1 485 406 (1 331 597 to 1 698 842)	9·1 (−6·0 to 59·7)
Middle SDI	78 203 (68 105 to 86 695)	−3·4 (−12·3 to 19·1)	105 724 (92 431 to 114 403)	26·1 (16·4 to 51·3)	2 714 483 (2 381 230 to 3 017 478)	−13·4 (−22·2 to 5·2)
High-middle SDI	48 091 (42 491 to 51 893)	−2·5 (−11·0 to 6·8)	79 703 (72 810 to 85 313)	36·9 (28·1 to 49·8)	1 564 401 (1 389 182 to 1 686 138)	−10·5 (−18·4 to −1·4)
High SDI	54 526 (43 837 to 57 711)	−4·6 (−25·9 to 1·7)	92 681 (74 397 to 99 558)	22·0 (−5·9 to 32·2)	1 443 970 (1 219 370 to 1 577 324)	−10·0 (−27·1 to −0·1)
**High-income North America**	**18 885 (16 471 to 20 054)**	**−7·1 (−17·6 to 4·6)**	**28 239 (25 257 to 30 711)**	**15·3 (4·9 to 33·4)**	**509 907 (455 284 to 556 592)**	**−10·8 (−18·9 to 2·4)**
Canada	2104 (1737 to 2356)	−9·9 (−24·6 to 6·3)	3501 (2801 to 3952)	14·4 (−5·8 to 34·8)	56 379 (45 459 to 63 690)	−12·2 (−26·1 to 3·9)
Greenland	2 (1 to 4)	−25·4 (−47·4 to 6·1)	2 (2 to 4)	−17·5 (−37·2 to 8·5)	68 (43 to 123)	−30·9 (−53·0 to 3·1)
USA	16 779 (14 745 to 17 756)	−6·8 (−16·7 to 4·3)	24 725 (22 447 to 26 908)	15·2 (6·0 to 33·3)	453 457 (410 642 to 491 397)	−10·7 (−18·3 to 2·4)
**Australasia**	**1707 (1341 to 1901)**	**−10·6 (−31·3 to 3·3)**	**2088 (1649 to 2320)**	**1·4 (−22·3 to 20·2)**	**46 523 (37 875 to 53 362)**	**−16·2 (−32·5 to −0·2)**
Australia	1426 (1111 to 1595)	−10·3 (−30·3 to 4·0)	1759 (1394 to 1961)	1·5 (−21·0 to 20·5)	38 597 (31 142 to 44 041)	−15·9 (−31·2 to 0·8)
New Zealand	282 (223 to 326)	−11·9 (−36·0 to 3·8)	329 (254 to 370)	1·0 (−27·7 to 16·6)	7925 (6390 to 9195)	−17·2 (−38·0 to −0·9)
**High-income Asia Pacific**	**4027 (3447 to 4523)**	**−8·6 (−28·4 to 4·7)**	**12 817 (10 719 to 13 993)**	**16·4 (−10·1 to 27·8)**	**110 751 (96 338 to 128 371)**	**−12·7 (−32·6 to 0·6)**
Brunei	12 (10 to 16)	23·0 (−5·3 to 62·3)	31 (25 to 39)	127·0 (77·6 to 189·5)	506 (399 to 659)	22·2 (−5·4 to 62·8)
Japan	2619 (2059 to 2845)	−5·7 (−27·2 to 5·1)	8953 (6838 to 9761)	1·7 (−26·0 to 11·5)	67 929 (56 427 to 76 950)	−7·8 (−27·9 to 4·6)
Singapore	74 (57 to 94)	−14·8 (−42·3 to 12·0)	216 (175 to 277)	98·7 (36·0 to 162·9)	2393 (1870 to 3104)	−22·0 (−46·9 to 6·5)
South Korea	1321 (946 to 1792)	−23·7 (−47·3 to 8·0)	3617 (2962 to 4632)	74·5 (22·4 to 135·9)	39 924 (28 701 to 56 772)	−26·4 (−49·0 to 4·6)
**Western Europe**	**28 201 (20 814 to 30 453)**	**1·0 (−29·8 to 10·3)**	**48 838 (36 877 to 54 037)**	**32·5 (−10·1 to 48·8)**	**721 787 (574 403 to 798 010)**	**−7·8 (−32·9 to 0·8)**
Andorra	6 (4 to 7)	2·9 (−23·8 to 40·6)	13 (10 to 17)	19·3 (−7·1 to 57·6)	151 (116 to 196)	−2·1 (−25·9 to 31·1)
Austria	520 (389 to 590)	−4·5 (−34·2 to 8·6)	804 (607 to 993)	18·4 (−23·6 to 51·2)	13 708 (10 910 to 15 910)	−12·6 (−38·0 to 0·4)
Belgium	676 (567 to 850)	−31·7 (−43·5 to 1·2)	1454 (1147 to 1898)	−15·1 (−37·8 to 32·6)	18 041 (15 271 to 23 772)	−32·2 (−44·0 to −0·9)
Cyprus	48 (42 to 55)	−2·9 (−16·6 to 16·7)	88 (72 to 111)	77·3 (40·3 to 123·8)	1352 (1198 to 1603)	−3·6 (−16·7 to 16·6)
Denmark	491 (377 to 569)	−11·7 (−37·2 to 5·3)	1495 (1173 to 1715)	39·2 (−3·5 to 68·0)	12 554 (10 246 to 14 536)	−18·1 (−41·0 to −0·8)
Finland	356 (294 to 418)	−10·4 (−39·9 to 5·0)	984 (785 to 1197)	15·0 (−24·2 to 43·6)	9154 (7673 to 11 419)	−15·8 (−42·8 to 0·1)
France	3570 (2492 to 4053)	6·6 (−27·6 to 23·3)	6359 (4669 to 8038)	37·8 (−12·3 to 79·9)	94 168 (70 656 to 105 758)	−0·2 (−30·7 to 14·8)
Germany	6104 (4487 to 6938)	5·1 (−35·9 to 24·1)	8300 (6013 to 9781)	32·0 (−17·6 to 63·3)	150 993 (117 742 to 172 869)	−8·2 (−38·2 to 6·7)
Greece	1210 (946 to 1335)	3·7 (−12·9 to 14·8)	1902 (1520 to 2514)	40·1 (8·4 to 76·8)	28 507 (25 035 to 33 956)	−3·0 (−16·4 to 8·2)
Iceland	30 (24 to 33)	6·8 (−26·1 to 24·7)	79 (61 to 95)	56·6 (6·7 to 102·0)	835 (694 to 947)	1·6 (−29·3 to 17·7)
Ireland	275 (224 to 340)	−9·3 (−31·4 to 11·6)	571 (443 to 726)	55·8 (14·1 to 100·3)	7878 (6441 to 10 399)	−13·2 (−32·0 to 7·8)
Israel	446 (309 to 548)	24·2 (−32·0 to 63·6)	604 (442 to 715)	44·3 (−21·9 to 84·6)	13 150 (9995 to 16 187)	15·9 (−33·1 to 48·4)
Italy	4057 (2867 to 4625)	−10·9 (−31·9 to 3·0)	8464 (5777 to 10 767)	39·3 (0·2 to 77·4)	97 950 (76 259 to 113 932)	−18·6 (−34·8 to −0·9)
Luxembourg	39 (31 to 47)	−14·1 (−40·8 to 3·7)	115 (88 to 147)	11·2 (−23·6 to 48·4)	1089 (887 to 1357)	−21·1 (−45·7 to −1·6)
Malta	26 (20 to 32)	0·9 (−26·2 to 26·1)	40 (31 to 50)	48·1 (2·6 to 86·9)	695 (532 to 867)	2·4 (−25·2 to 26·6)
Netherlands	988 (680 to 1 137)	14·3 (−32·2 to 35·9)	2427 (1645 to 2970)	56·9 (−7·0 to 106·8)	27 553 (18 781 to 31 675)	6·9 (−34·7 to 25·4)
Norway	361 (276 to 414)	3·6 (−33·2 to 22·5)	1114 (853 to 1284)	41·3 (−8·1 to 73·4)	9925 (7957 to 11 419)	−3·8 (−36·1 to 12·5)
Portugal	857 (574 to 973)	17·2 (−30·4 to 38·6)	1373 (955 to 1 767)	68·4 (−4·9 to 120·1)	21 568 (15 760 to 24 422)	1·0 (−37·6 to 17·9)
Spain	2901 (2100 to 3272)	5·8 (−32·2 to 20·8)	5054 (3693 to 6500)	56·6 (0·3 to 99·2)	73 260 (58 060 to 88 757)	−5·0 (−36·4 to 9·0)
Sweden	604 (505 to 700)	−11·7 (−31·4 to 4·7)	1556 (1312 to 1859)	−2·8 (−25·8 to 19·0)	16 035 (13 556 to 18 917)	−16·9 (−33·5 to −0·4)
Switzerland	445 (310 to 583)	8·1 (−37·9 to 47·2)	942 (687 to 1162)	14·1 (−39·4 to 56·4)	11 537 (8410 to 15 111)	1·4 (−42·8 to 38·0)
UK	4194 (3163 to 4432)	6·5 (−25·7 to 13·8)	5053 (3866 to 5377)	21·6 (−12·9 to 30·8)	111 667 (89 431 to 117 828)	−2·9 (−27·1 to 5·4)
**Southern Latin America**	**2039 (1784 to 2275)**	**8·5 (−18·3 to 29·8)**	**2272 (2050 to 2436)**	**15·3 (−14·7 to 33·9)**	**62 394 (55 182 to 69 698)**	**3·0 (−23·7 to 22·5)**
Argentina	1427 (1237 to 1596)	10·5 (−16·8 to 33·1)	1570 (1402 to 1702)	14·0 (−12·3 to 33·6)	43 606 (38 536 to 48 645)	4·2 (−22·1 to 24·5)
Chile	468 (356 to 591)	15·8 (−32·3 to 64·7)	550 (491 to 597)	33·3 (−18·1 to 68·4)	14 663 (11 299 to 18 414)	7·9 (−38·1 to 52·7)
Uruguay	144 (124 to 161)	4·6 (−17·0 to 20·4)	152 (133 to 165)	12·7 (−11·8 to 27·9)	4125 (3555 to 4589)	0·9 (−19·9 to 16·5)
**Eastern Europe**	**10 719 (8459 to 13 555)**	**−4·6 (−24·6 to 19·6)**	**14 538 (12 762 to 16 177)**	**5·3 (−4·0 to 21·7)**	**350 274 (279 400 to 441 568)**	**−11·3 (−28·6 to 13·4)**
Belarus	450 (359 to 545)	8·2 (−16·6 to 31·9)	600 (502 to 670)	23·6 (2·5 to 41·2)	14 823 (11 649 to 17 799)	0·9 (−23·9 to 23·1)
Estonia	82 (50 to 99)	17·3 (−30·0 to 44·9)	151 (91 to 182)	76·3 (0·2 to 114·3)	2279 (1487 to 2730)	−2·8 (−37·7 to 18·1)
Latvia	142 (95 to 170)	45·0 (−24·1 to 86·9)	203 (135 to 237)	74·9 (−9·6 to 116·7)	3953 (2809 to 4683)	27·2 (−32·4 to 63·4)
Lithuania	206 (140 to 239)	39·5 (−7·4 to 65·7)	341 (227 to 398)	88·2 (23·2 to 121·2)	5837 (4301 to 6701)	24·3 (−12·7 to 45·3)
Moldova	173 (143 to 201)	4·6 (−19·0 to 22·9)	191 (166 to 217)	−0·1 (−23·9 to 9·6)	6074 (4872 to 7006)	−7·8 (−30·7 to 11·5)
Russia	7469 (5421 to 10 134)	−8·8 (−33·1 to 26·6)	10 072 (8789 to 11 193)	0·5 (−10·6 to 23·6)	243 185 (179 921 to 325 252)	−15·2 (−37·1 to 19·5)
Ukraine	2197 (1692 to 2970)	−0·5 (−24·2 to 29·3)	2979 (2570 to 3660)	7·3 (−7·1 to 19·9)	74 123 (57 240 to 98 013)	−5·4 (−28·3 to 21·2)
**Central Europe**	**9332 (7421 to 10 173)**	**14·0 (−15·3 to 25·8)**	**10 656 (8335 to 11 508)**	**24·7 (−7·2 to 37·2)**	**259 460 (215 615 to 280 249)**	**−1·2 (−21·6 to 7·5)**
Albania	246 (186 to 293)	40·7 (−4·5 to 83·4)	251 (206 to 282)	37·2 (1·2 to 74·4)	8009 (6403 to 9318)	36·6 (−4·8 to 76·6)
Bosnia and Herzegovina	390 (296 to 484)	17·7 (−11·0 to 51·2)	398 (316 to 494)	16·8 (1·7 to 51·2)	10 924 (8651 to 13 733)	6·9 (−15·0 to 36·9)
Bulgaria	639 (429 to 784)	31·7 (−12·6 to 64·0)	730 (491 to 834)	40·2 (−3·7 to 61·3)	18 142 (12 401 to 22 130)	15·3 (−15·6 to 38·9)
Croatia	434 (308 to 518)	19·1 (−25·9 to 47·2)	753 (525 to 927)	54·5 (−4·4 to 99·0)	10 873 (8501 to 12 714)	2·4 (−30·9 to 24·1)
Czech Republic	739 (574 to 838)	1·4 (−41·4 to 21·4)	772 (585 to 869)	6·6 (−39·0 to 26·6)	19 949 (15 975 to 23 014)	−13·1 (−44·6 to 1·7)
Hungary	761 (645 to 989)	−11·8 (−27·8 to 5·3)	841 (736 to 1113)	−4·9 (−21·2 to 21·7)	20 617 (17 194 to 28 224)	−21·9 (−37·6 to 1·8)
Macedonia	181 (130 to 209)	20·8 (−5·5 to 42·1)	190 (140 to 215)	24·0 (1·7 to 40·3)	5420 (4163 to 6155)	10·0 (−7·6 to 26·4)
Montenegro	51 (44 to 63)	0·7 (−13·5 to 18·3)	57 (50 to 67)	6·3 (−3·9 to 19·6)	1553 (1330 to 1938)	−5·5 (−18·9 to 8·9)
Poland	3106 (2262 to 3568)	7·3 (−18·7 to 23·4)	3485 (2584 to 3887)	19·6 (−9·2 to 37·8)	84 181 (67 409 to 95 638)	−8·9 (−26·6 to 12·9)
Romania	1473 (913 to 1730)	39·8 (−20·1 to 69·2)	1645 (1047 to 1866)	46·9 (−10·8 to 72·0)	42 361 (27 106 to 49 439)	14·3 (−30·8 to 34·6)
Serbia	792 (648 to 897)	10·6 (−5·0 to 29·2)	925 (754 to 1 052)	20·6 (6·1 to 41·1)	22 658 (19 144 to 25 313)	0·5 (−11·6 to 15·6)
Slovakia	383 (307 to 450)	17·3 (−6·9 to 41·3)	448 (366 to 500)	36·3 (13·4 to 58·4)	11 179 (9278 to 13 057)	7·2 (−9·1 to 25·2)
Slovenia	136 (82 to 166)	11·6 (−39·8 to 41·0)	161 (94 to 188)	30·2 (−32·9 to 57·6)	3594 (2269 to 4382)	−2·3 (−44·3 to 20·7)
**Central Asia**	**3064 (2586 to 3358)**	**20·4 (4·1 to 38·0)**	**3619 (3039 to 3860)**	**19·8 (5·7 to 33·3)**	**127 439 (106 135 to 140 171)**	**16·1 (−0·1 to 32·5)**
Armenia	210 (167 to 245)	4·3 (−19·7 to 28·2)	233 (190 to 256)	4·1 (−18·9 to 22·2)	6830 (5622 to 7922)	−2·2 (−30·7 to 21·1)
Azerbaijan	423 (337 to 525)	−0·6 (−20·5 to 20·5)	499 (441 to 578)	−0·1 (−12·2 to 9·5)	16 838 (13 586 to 20 700)	−2·7 (−22·3 to 18·0)
Georgia	196 (129 to 247)	54·6 (−5·2 to 111·2)	206 (141 to 231)	46·8 (−13·2 to 80·4)	6452 (4299 to 8094)	42·1 (−7·0 to 92·4)
Kazakhstan	527 (426 to 631)	−4·9 (−23·9 to 20·7)	637 (555 to 694)	−1·4 (−12·4 to 15·0)	20 931 (16 780 to 24 827)	−9·5 (−27·0 to 16·4)
Kyrgyzstan	126 (86 to 149)	31·8 (−13·1 to 60·9)	145 (98 to 166)	26·7 (−23·6 to 48·6)	5296 (3656 to 6241)	26·5 (−21·8 to 56·1)
Mongolia	76 (54 to 93)	79·6 (−13·6 to 179·8)	83 (63 to 97)	84·8 (−0·1 to 175·4)	3015 (2378 to 3763)	66·1 (−11·7 to 150·8)
Tajikistan	203 (143 to 262)	16·4 (−9·3 to 48·0)	232 (166 to 282)	11·6 (−9·5 to 34·2)	9486 (7078 to 12 057)	11·2 (−15·4 to 42·4)
Turkmenistan	189 (157 to 249)	37·7 (0·5 to 78·8)	223 (187 to 293)	41·1 (1·7 to 82·8)	8564 (7030 to 11 169)	34·3 (−3·0 to 76·7)
Uzbekistan	1113 (860 to 1309)	40·0 (8·4 to 85·7)	1360 (1038 to 1509)	37·9 (12·7 to 76·2)	50 027 (36 549 to 59 480)	36·2 (5·6 to 79·5)
**Central Latin America**	**5384 (4490 to 5968)**	**24·8 (−7·8 to 41·2)**	**6183 (5174 to 6671)**	**28·7 (−5·3 to 43·0)**	**204 170 (175 723 to 229 524)**	**16·7 (−13·0 to 30·5)**
Colombia	1246 (962 to 1472)	20·9 (−18·7 to 45·2)	1412 (1116 to 1607)	24·2 (−17·7 to 42·4)	44 994 (35 554 to 54 787)	12·7 (−24·6 to 34·3)
Costa Rica	134 (116 to 156)	3·6 (−16·8 to 28·6)	149 (130 to 170)	6·7 (−12·4 to 28·0)	4617 (3964 to 5563)	2·6 (−17·4 to 28·4)
El Salvador	182 (120 to 223)	82·0 (−11·7 to 171·8)	197 (132 to 239)	91·8 (0·3 to 184·5)	6557 (4578 to 7915)	69·6 (−12·2 to 148·9)
Guatemala	276 (204 to 395)	−18·1 (−48·1 to 32·3)	306 (260 to 418)	−19·0 (−46·9 to 23·9)	11 812 (8817 to 18 190)	−21·6 (−52·9 to 33·5)
Honduras	176 (99 to 281)	28·0 (−18·4 to 97·0)	170 (108 to 243)	27·2 (0·1 to 75·8)	6421 (4372 to 9265)	16·8 (−20·0 to 71·8)
Mexico	2552 (2037 to 2804)	26·4 (−10·2 to 39·1)	3012 (2429 to 3274)	31·8 (−7·4 to 43·6)	97 707 (78 602 to 107 753)	17·8 (−16·6 to 29·2)
Nicaragua	119 (99 to 151)	15·4 (−11·7 to 66·5)	134 (120 to 154)	16·0 (−0·1 to 54·2)	5086 (4271 to 6172)	1·1 (−15·2 to 26·8)
Panama	108 (91 to 128)	10·9 (−17·6 to 39·1)	123 (108 to 137)	15·4 (−12·7 to 38·4)	4091 (3425 to 4905)	11·4 (−15·9 to 39·3)
Venezuela	592 (403 to 744)	65·4 (−5·8 to 120·3)	680 (473 to 766)	69·0 (5·1 to 100·8)	22 884 (16 604 to 28 966)	64·0 (0·2 to 120·4)
**Andean Latin America**	**1575 (1186 to 1874)**	**24·9 (−2·9 to 48·2)**	**1749 (1334 to 1975)**	**21·1 (−1·8 to 37·1)**	**59 906 (45 309 to 71 224)**	**16·3 (−7·6 to 36·9)**
Bolivia	294 (209 to 378)	35·5 (−3·1 to 88·1)	300 (235 to 350)	26·8 (0·3 to 58·4)	11 658 (8271 to 14 829)	25·9 (−3·1 to 66·2)
Ecuador	415 (349 to 467)	16·5 (−13·9 to 38·0)	472 (402 to 519)	18·6 (−13·2 to 37·7)	16 078 (13 543 to 18 588)	12·5 (−18·0 to 33·4)
Peru	866 (567 to 1 124)	26·2 (−9·0 to 63·8)	977 (667 to 1167)	20·9 (−4·7 to 44·5)	32 170 (21 468 to 41 221)	15·0 (−16·9 to 51·1)
**Caribbean**	**1273 (1089 to 1383)**	**12·9 (−4·7 to 27·5)**	**1487 (1285 to 1578)**	**14·3 (−2·2 to 25·5)**	**43 479 (38 567 to 47 120)**	**5·3 (−10·6 to 18·6)**
Antigua and Barbuda	2 (2 to 2)	6·1 (−16·2 to 27·7)	2 (2 to 2)	13·2 (−5·0 to 25·3)	66 (57 to 78)	7·0 (−13·9 to 28·7)
The Bahamas	10 (8 to 11)	−12·1 (−29·0 to 7·2)	11 (11 to 13)	−7·4 (−25·3 to 9·1)	341 (295 to 401)	−14·2 (−34·8 to 9·9)
Barbados	9 (8 to 12)	−8·6 (−27·1 to 14·1)	10 (10 to 13)	−3·8 (−21·0 to 13·6)	276 (243 to 346)	−10·5 (−28·5 to 13·3)
Belize	7 (5 to 8)	16·4 (−10·4 to 60·4)	7 (7 to 9)	9·3 (−8·1 to 41·3)	282 (228 to 358)	5·8 (−18·9 to 46·1)
Bermuda	2 (1 to 3)	74·7 (−46·3 to 143·3)	3 (1 to 3)	88·9 (−44·5 to 140·4)	65 (34 to 80)	49·8 (−50·2 to 104·3)
Cuba	632 (480 to 726)	22·4 (−14·8 to 48·0)	695 (536 to 775)	24·1 (−15·0 to 45·9)	18 598 (14 232 to 21 354)	12·5 (−23·1 to 36·5)
Dominica	1 (1 to 1)	6·6 (−16·8 to 41·7)	1 (1 to 1)	9·2 (−11·5 to 36·6)	40 (34 to 48)	6·4 (−16·5 to 42·8)
Dominican Republic	197 (159 to 247)	6·6 (−14·1 to 38·7)	216 (187 to 269)	8·8 (−3·3 to 30·1)	7636 (6063 to 10 876)	2·0 (−14·9 to 23·7)
Grenada	3 (3 to 4)	20·6 (−8·9 to 69·1)	3 (3 to 4)	19·1 (−2·2 to 56·4)	120 (99 to 144)	19·0 (−13·2 to 70·6)
Guyana	9 (8 to 11)	12·5 (−9·9 to 36·2)	10 (9 to 11)	11·2 (−8·6 to 27·8)	372 (306 to 441)	9·7 (−14·7 to 32·3)
Haiti	191 (130 to 249)	20·1 (−4·6 to 49·8)	197 (143 to 232)	8·6 (−4·0 to 27·0)	8679 (6274 to 11 021)	12·0 (−14·2 to 42·9)
Jamaica	56 (44 to 69)	19·5 (−9·5 to 78·5)	60 (49 to 69)	12·2 (−7·0 to 61·1)	2169 (1614 to 2759)	13·9 (−14·1 to 64·2)
Puerto Rico	97 (78 to 110)	−15·1 (−30·7 to 11·2)	139 (118 to 152)	11·3 (−9·5 to 54·6)	2661 (2284 to 2997)	−22·3 (−38·5 to 7·5)
Saint Lucia	4 (3 to 4)	7·9 (−15·9 to 33·6)	4 (4 to 4)	10·1 (−13·6 to 35·3)	122 (108 to 143)	4·8 (−20·0 to 34·1)
Saint Vincent and the Grenadines	3 (2 to 3)	66·1 (1·6 to 108·1)	3 (3 to 3)	67·7 (4·3 to 101·7)	100 (87 to 114)	65·2 (−2·1 to 111·7)
Suriname	21 (18 to 25)	12·4 (−6·9 to 64·5)	24 (21 to 28)	12·5 (−4·8 to 58·3)	825 (693 to 992)	6·7 (−12·5 to 50·5)
Trinidad and Tobago	26 (23 to 30)	2·9 (−22·5 to 24·7)	30 (27 to 34)	9·7 (−17·9 to 27·2)	995 (839 to 1161)	3·7 (−24·5 to 32·8)
Virgin Islands	4 (3 to 5)	29·3 (−13·5 to 74·8)	6 (5 to 7)	86·4 (37·6 to 137·8)	112 (88 to 135)	22·3 (−18·0 to 64·7)
**Tropical Latin America**	**9523 (6812 to 10 453)**	**78·5 (−9·1 to 112·8)**	**10 653 (7702 to 11 510)**	**79·8 (−9·2 to 110·6)**	**319 741 (240 591 to 350 958)**	**65·3 (−10·8 to 96·1)**
Brazil	9402 (6635 to 10 338)	79·5 (−11·5 to 114·5)	10 521 (7572 to 11 369)	80·8 (−11·2 to 112·1)	315 161 (233 496 to 346 087)	66·4 (−12·6 to 97·9)
Paraguay	120 (82 to 213)	23·7 (−14·7 to 273·4)	132 (94 to 230)	20·6 (−14·1 to 253·0)	4580 (3207 to 7852)	16·2 (−19·0 to 232·5)
**East Asia**	**60 641 (54 294 to 68 081)**	**−19·4 (−28·0 to −1·6)**	**108 444 (98 490 to 121 560)**	**47·0 (31·6 to 78·1)**	**1 986 794 (1 793 427 to 2 244 266)**	**−27·9 (−36·5 to −8·3)**
China	59 120 (53 264 to 66 813)	−19·8 (−28·6 to −1·9)	106 207 (96 980 to 119 885)	47·1 (31·2 to 78·8)	1 933 243 (1 756 995 to 2 196 524)	−28·6 (−37·3 to −8·4)
North Korea	937 (565 to 1217)	14·2 (−2·9 to 34·4)	906 (529 to 1144)	2·6 (−7·7 to 16·2)	35 103 (21 397 to 45 871)	12·9 (−5·3 to 36·0)
Taiwan (province of China)	583 (412 to 710)	−4·7 (−23·1 to 13·7)	1330 (929 to 1594)	117·4 (69·3 to 159·9)	18 448 (12 638 to 22 268)	−4·2 (−21·4 to 14·0)
**Southeast Asia**	**14 196 (10 685 to 16 783)**	**20·6 (2·7 to 53·3)**	**15 540 (11 650 to 18 228)**	**15·7 (1·6 to 40·0)**	**532 546 (410 786 to 631 487)**	**10·0 (−1·6 to 35·6)**
Cambodia	276 (216 to 331)	33·4 (8·6 to 94·3)	263 (208 to 307)	8·9 (−5·7 to 45·9)	11 411 (9042 to 13 568)	27·5 (5·2 to 75·3)
Indonesia	5405 (3822 to 7431)	34·4 (15·7 to 70·7)	6337 (4442 to 8405)	14·6 (3·6 to 42·7)	214 521 (154 655 to 299 149)	21·1 (7·2 to 45·2)
Laos	113 (87 to 134)	35·6 (11·8 to 67·5)	113 (92 to 129)	6·4 (−3·4 to 23·5)	5481 (4250 to 6691)	33·9 (10·8 to 64·8)
Malaysia	431 (352 to 628)	8·0 (−20·2 to 91·7)	598 (505 to 786)	35·9 (5·4 to 119·9)	16 258 (13 494 to 21 269)	3·7 (−22·0 to 80·0)
Maldives	4 (3 to 5)	−7·3 (−31·0 to 25·6)	5 (4 to 5)	−6·8 (−25·4 to 11·2)	143 (114 to 182)	−23·5 (−46·4 to 7·9)
Mauritius	22 (18 to 29)	−8·9 (−27·0 to 16·0)	26 (23 to 34)	5·1 (−10·6 to 33·1)	742 (604 to 989)	−11·2 (−28·6 to 17·8)
Myanmar	1580 (1215 to 1861)	22·2 (−2·1 to 68·3)	1121 (893 to 1251)	10·7 (−4·0 to 40·3)	59 451 (46 771 to 70 526)	15·3 (−7·5 to 53·7)
Philippines	1969 (1625 to 2378)	−1·6 (−20·3 to 27·8)	2297 (2002 to 2623)	−2·8 (−18·5 to 22·3)	82 021 (68 724 to 99 990)	−9·5 (−28·6 to 22·3)
Sri Lanka	501 (352 to 681)	43·1 (−16·0 to 121·9)	534 (408 to 649)	53·8 (4·0 to 119·3)	15 774 (11 950 to 20 751)	33·7 (−14·8 to 95·8)
Seychelles	4 (3 to 5)	−15·7 (−33·3 to 22·0)	5 (4 to 6)	−3·4 (−20·7 to 34·3)	132 (108 to 176)	−19·3 (−37·9 to 15·2)
Thailand	2490 (1494 to 3105)	11·5 (−11·8 to 45·3)	2747 (1692 to 3299)	25·3 (7·7 to 45·5)	75 920 (47 576 to 93 389)	−4·0 (−20·3 to 24·3)
Timor-Leste	18 (12 to 23)	29·2 (−7·3 to 81·7)	18 (13 to 22)	2·7 (−8·7 to 15·9)	771 (513 to 1012)	19·1 (−17·2 to 59·6)
Vietnam	1384 (1069 to 1678)	8·2 (−15·5 to 45·5)	1452 (1160 to 1659)	10·8 (−1·3 to 31·1)	49 913 (37 580 to 60 883)	3·2 (−19·2 to 32·5)
**Oceania**	**108 (83 to 131)**	**6·0 (−13·4 to 59·7)**	**133 (108 to 154)**	**6·9 (−6·7 to 51·3)**	**4996 (3901 to 6065)**	**8·0 (−12·7 to 59·5)**
American Samoa	1 (1 to 1)	7·7 (−17·9 to 64·7)	1 (1 to 2)	22·6 (2·3 to 79·1)	47 (37 to 58)	5·2 (−18·8 to 59·1)
Federated States of Micronesia	2 (1 to 2)	21·0 (−19·9 to 135·4)	1 (1 to 2)	22·5 (−6·3 to 117·5)	63 (46 to 92)	19·4 (−21·4 to 122·8)
Fiji	13 (9 to 17)	9·2 (−30·6 to 134·0)	15 (12 to 18)	18·6 (−14·6 to 145·5)	534 (368 to 714)	16·4 (−27·4 to 154·1)
Guam	3 (2 to 4)	9·8 (−14·0 to 50·2)	4 (4 to 5)	31·0 (8·6 to 77·7)	101 (82 to 124)	11·3 (−13·6 to 53·2)
Kiribati	2 (1 to 2)	29·5 (−3·1 to 93·6)	1 (1 to 2)	14·9 (−8·0 to 59·4)	77 (57 to 109)	32·1 (−1·3 to 94·7)
Marshall Islands	1 (1 to 1)	0·5 (−23·8 to 55·3)	1 (1 to 1)	14·4 (−7·3 to 73·0)	38 (30 to 53)	2·2 (−22·3 to 52·4)
Northern Mariana Islands	1 (1 to 2)	5·6 (−23·0 to 55·8)	2 (2 to 3)	27·2 (8·0 to 68·6)	53 (41 to 68)	0·8 (−27·7 to 49·8)
Papua New Guinea	72 (47 to 94)	5·5 (−16·6 to 50·1)	82 (58 to 99)	3·7 (−8·1 to 33·8)	3420 (2345 to 4469)	5·2 (−18·1 to 45·9)
Samoa	3 (2 to 4)	16·6 (−17·2 to 82·6)	3 (2 to 4)	18·2 (−8·2 to 75·3)	134 (90 to 188)	14·4 (−18·0 to 73·0)
Solomon Islands	7 (5 to 9)	17·1 (−9·6 to 79·3)	6 (5 to 7)	7·5 (−8·2 to 56·7)	299 (208 to 390)	22·9 (−6·6 to 84·6)
Tonga	2 (1 to 2)	13·3 (−18·3 to 75·3)	2 (2 to 2)	13·2 (−7·9 to 65·9)	80 (60 to 101)	17·2 (−15·8 to 83·7)
Vanuatu	3 (2 to 4)	19·3 (−10·0 to 111·8)	3 (2 to 4)	10·0 (−8·3 to 91·5)	145 (98 to 192)	25·1 (−7·0 to 117·7)
**North Africa and Middle East**	**16 155 (13 304 to 18 613)**	**18·1 (−5·4 to 78·4)**	**18 449 (15 251 to 20 751)**	**20·5 (2·0 to 75·8)**	**629 780 (512 858 to 721 066)**	**12·1 (−5·4 to 55·2)**
Afghanistan	627 (443 to 766)	27·2 (3·0 to 96·9)	574 (403 to 673)	10·9 (−1·5 to 58·4)	29 461 (21 647 to 35 772)	29·0 (5·9 to 91·4)
Algeria	674 (491 to 887)	30·3 (−2·4 to 89·4)	663 (482 to 848)	26·1 (1·2 to 73·7)	26 033 (17 546 to 34 543)	27·4 (−2·6 to 74·9)
Bahrain	19 (15 to 24)	−11·4 (−32·9 to 20·8)	22 (20 to 26)	−8·5 (−21·7 to 14·8)	718 (563 to 912)	−13·4 (−34·7 to 19·2)
Egypt	2019 (1402 to 3145)	19·6 (−11·3 to 83·5)	2377 (1828 to 3595)	24·0 (0·9 to 74·0)	84 050 (63 080 to 120 342)	11·4 (−13·8 to 55·5)
Iran	3307 (2182 to 4267)	41·5 (−8·8 to 196·7)	3926 (2715 to 4557)	42·3 (5·5 to 186·8)	120 535 (80 795 to 154 063)	30·3 (−11·3 to 152·4)
Iraq	1187 (935 to 1475)	14·4 (−14·8 to 64·4)	1226 (1049 to 1358)	12·3 (−2·2 to 46·5)	54 200 (42 124 to 67 392)	13·9 (−15·2 to 62·2)
Jordan	177 (135 to 225)	18·0 (−19·6 to 106·8)	204 (167 to 230)	23·3 (1·0 to 91·9)	7677 (5878 to 9725)	11·6 (−23·1 to 87·1)
Kuwait	50 (35 to 72)	3·7 (−24·9 to 48·0)	74 (59 to 102)	20·3 (3·0 to 65·6)	2104 (1502 to 3016)	−0·9 (−26·3 to 44·8)
Lebanon	200 (166 to 244)	−25·5 (−43·6 to 2·2)	302 (256 to 336)	0·3 (−18·3 to 16·4)	7381 (5965 to 8887)	−28·4 (−46·1 to −0·7)
Libya	212 (137 to 298)	22·8 (−16·3 to 139·2)	245 (163 to 333)	42·2 (2·7 to 162·0)	8080 (5109 to 11 096)	18·0 (−17·4 to 113·8)
Morocco	686 (543 to 864)	36·2 (3·5 to 119·2)	761 (588 to 924)	17·7 (−6·2 to 86·9)	26 240 (18 756 to 33 557)	30·7 (3·0 to 98·5)
Oman	64 (47 to 116)	55·7 (−11·1 to 540·1)	79 (59 to 145)	70·1 (2·7 to 569·6)	2602 (1903 to 4579)	46·2 (−13·8 to 436·4)
Palestine	211 (181 to 240)	19·2 (−11·2 to 72·5)	227 (195 to 259)	21·5 (0·2 to 67·4)	9403 (8068 to 11 171)	9·5 (−15·1 to 49·1)
Qatar	26 (19 to 35)	−20·5 (−51·6 to 57·2)	33 (29 to 38)	−10·1 (−31·5 to 68·4)	1142 (853 to 1 507)	−27·6 (−55·0 to 39·1)
Saudi Arabia	731 (584 to 1002)	42·6 (−13·5 to 405·0)	948 (800 to 1255)	50·7 (−5·2 to 436·1)	26 182 (20 275 to 37 082)	28·9 (−17·6 to 290·8)
Sudan	772 (655 to 888)	32·2 (9·0 to 107·8)	844 (729 to 964)	12·2 (−3·3 to 59·5)	35 549 (28 850 to 44 383)	30·5 (8·0 to 95·9)
Syria	502 (408 to 638)	21·1 (−12·9 to 146·5)	544 (448 to 708)	18·3 (−10·6 to 133·2)	18 915 (15 613 to 25 113)	15·6 (−15·8 to 114·0)
Tunisia	273 (213 to 353)	13·7 (−19·6 to 103·7)	293 (229 to 358)	15·7 (−9·9 to 98·9)	10 102 (7278 to 13 069)	6·0 (−22·4 to 74·1)
Turkey	3686 (2878 to 4478)	−1·0 (−20·9 to 29·2)	4301 (3513 to 4877)	9·7 (−2·1 to 31·2)	126 472 (97 715 to 153 502)	−8·0 (−23·9 to 13·2)
United Arab Emirates	260 (163 to 447)	48·7 (−11·2 to 211·9)	283 (203 to 461)	48·2 (4·6 to 198·6)	11 444 (7288 to 19 244)	38·3 (−16·5 to 186·0)
Yemen	473 (362 to 576)	36·5 (1·5 to 181·5)	506 (402 to 583)	21·2 (−0·7 to 128·2)	21 482 (16 930 to 26 211)	31·2 (0·2 to 145·1)
**South Asia**	**27 617 (24 899 to 33 462)**	**20·5 (−0·3 to 118·0)**	**31 212 (28 628 to 37 138)**	**5·2 (−10·5 to 89·4)**	**1 114 836 (1 011 135 to 1 343 880)**	**13·6 (−6·4 to 99·8)**
Bangladesh	2004 (1226 to 2721)	−25·6 (−42·3 to 1·5)	2510 (1534 to 3367)	−9·2 (−28·1 to 18·3)	86 006 (52 948 to 117 815)	−26·6 (−45·8 to −5·5)
Bhutan	12 (7 to 19)	4·3 (−22·5 to 92·6)	12 (7 to 17)	−0·2 (−17·1 to 74·9)	522 (291 to 802)	−2·2 (−27·8 to 70·4)
India	21 042 (18 847 to 25 993)	23·4 (1·4 to 132·7)	23 344 (21 446 to 28 329)	3·3 (−12·3 to 93·4)	811 288 (731 493 to 1 008 612)	14·0 (−7·6 to 112·2)
Nepal	412 (220 to 623)	13·2 (−9·3 to 81·0)	425 (221 to 607)	−4·7 (−19·0 to 41·2)	16 459 (8469 to 25 742)	3·7 (−19·4 to 57·0)
Pakistan	4146 (3186 to 5448)	40·7 (−3·3 to 174·2)	4922 (3690 to 6201)	25·6 (−7·5 to 135·6)	200 561 (146 729 to 262 906)	38·9 (−2·0 to 152·1)
**Southern sub-Saharan Africa**	**1177 (982 to 1317)**	**27·6 (4·5 to 94·6)**	**1292 (1083 to 1388)**	**17·7 (1·5 to 74·1)**	**50 339 (40 967 to 55 989)**	**31·0 (5·9 to 91·1)**
Botswana	30 (15 to 49)	30·8 (−38·2 to 179·2)	32 (26 to 41)	28·1 (−0·7 to 126·6)	1196 (620 to 1856)	26·9 (−37·7 to 173·7)
Lesotho	25 (17 to 37)	38·7 (−18·6 to 242·6)	29 (22 to 39)	37·8 (−3·2 to 212·2)	1036 (749 to 1485)	40·3 (−17·3 to 250·7)
Namibia	23 (15 to 30)	16·5 (−29·1 to 101·0)	24 (19 to 27)	16·6 (−4·1 to 65·6)	951 (664 to 1231)	14·8 (−27·3 to 91·4)
South Africa	822 (687 to 915)	27·9 (−0·4 to 83·5)	967 (804 to 1 049)	25·9 (2·4 to 75·7)	34 233 (26 639 to 39 366)	35·9 (−0·6 to 88·2)
Swaziland	14 (9 to 20)	14·4 (−27·3 to 108·1)	16 (12 to 20)	16·3 (−2·7 to 81·8)	623 (414 to 882)	18·3 (−22·2 to 113·3)
Zimbabwe	263 (206 to 333)	39·2 (1·2 to 154·5)	224 (192 to 256)	−1·9 (−17·5 to 72·9)	12 300 (9744 to 15 435)	34·4 (−1·2 to 137·5)
**Western sub-Saharan Africa**	**5238 (4210 to 6459)**	**25·4 (4·8 to 53·6)**	**5468 (4268 to 6635)**	**16·3 (0·5 to 32·6)**	**245 490 (189 605 to 310 117)**	**27·3 (5·2 to 47·3)**
Benin	158 (124 to 183)	30·1 (−1·3 to 77·7)	146 (115 to 165)	25·6 (0·4 to 56·0)	7028 (5377 to 8265)	31·1 (−2·1 to 66·0)
Burkina Faso	237 (180 to 276)	27·0 (−6·6 to 71·2)	222 (170 to 260)	25·6 (−0·4 to 61·5)	11 393 (8399 to 13 764)	34·6 (−2·8 to 76·9)
Cameroon	432 (264 to 699)	33·9 (−4·3 to 82·1)	385 (248 to 607)	28·3 (0·4 to 59·0)	19 900 (11 947 to 32 581)	40·8 (−0·3 to 84·2)
Cape Verde	11 (9 to 16)	31·2 (−8·1 to 139·7)	11 (10 to 16)	19·3 (−10·0 to 111·4)	487 (407 to 598)	26·6 (−6·0 to 121·5)
Chad	162 (125 to 193)	23·0 (−3·0 to 69·1)	162 (125 to 188)	22·5 (0·3 to 54·8)	8040 (6101 to 9625)	29·7 (−0·3 to 66·5)
Côte d'Ivoire	264 (214 to 316)	27·7 (−2·6 to 92·9)	221 (186 to 249)	16·6 (−6·3 to 66·2)	11 271 (9314 to 13 242)	28·1 (−0·5 to 89·2)
The Gambia	18 (15 to 22)	17·6 (−7·0 to 54·6)	17 (14 to 20)	8·4 (−7·1 to 30·8)	827 (689 to 983)	14·5 (−8·7 to 44·3)
Ghana	652 (551 to 769)	24·2 (−2·1 to 74·1)	608 (522 to 676)	5·8 (−11·5 to 46·8)	29 701 (23 563 to 35 300)	20·7 (−3·0 to 65·4)
Guinea	193 (153 to 250)	21·5 (−8·6 to 68·8)	167 (142 to 201)	12·9 (−7·7 to 46·7)	7153 (5876 to 9138)	18·7 (−7·0 to 58·5)
Guinea-Bissau	31 (23 to 38)	23·7 (−3·3 to 61·0)	26 (20 to 31)	20·1 (0·1 to 38·5)	1382 (1045 to 1657)	26·6 (−1·7 to 58·6)
Liberia	56 (45 to 66)	28·9 (2·3 to 58·5)	57 (47 to 65)	18·5 (−1·4 to 36·3)	2409 (1914 to 2868)	27·7 (1·9 to 55·9)
Mali	154 (124 to 193)	7·8 (−14·7 to 35·3)	159 (142 to 185)	2·2 (−8·3 to 12·7)	6968 (5578 to 9098)	3·2 (−18·2 to 26·0)
Mauritania	64 (44 to 84)	21·2 (−11·5 to 59·4)	56 (44 to 66)	15·6 (−3·3 to 34·7)	2816 (2002 to 3586)	28·0 (−8·2 to 69·3)
Niger	206 (111 to 290)	18·3 (−11·0 to 54·0)	212 (116 to 276)	12·3 (−7·7 to 33·6)	9523 (5122 to 13 282)	18·2 (−13·9 to 59·4)
Nigeria	2165 (1542 to 3043)	22·9 (−4·2 to 53·2)	2632 (1938 to 3506)	15·8 (−0·1 to 33·5)	106 875 (73 141 to 156 444)	26·5 (−1·5 to 60·0)
São Tomé and Principe	2 (1 to 2)	22·2 (−11·4 to 75·9)	2 (1 to 2)	16·1 (−6·2 to 51·4)	78 (59 to 100)	16·0 (−11·7 to 56·7)
Senegal	231 (186 to 267)	36·8 (8·6 to 74·2)	190 (152 to 216)	20·8 (−1·2 to 40·9)	10 460 (8278 to 12 340)	39·7 (10·8 to 66·9)
Sierra Leone	88 (68 to 116)	33·6 (−4·5 to 89·5)	91 (69 to 117)	29·4 (−0·1 to 67·8)	4115 (3041 to 5544)	34·1 (−4·4 to 78·8)
Togo	114 (83 to 147)	36·0 (−2·1 to 100·1)	102 (76 to 127)	30·7 (−0·5 to 75·8)	5063 (3685 to 6422)	37·6 (−3·0 to 87·4)
**Eastern sub-Saharan Africa**	**4868 (4299 to 5911)**	**27·6 (5·5 to 72·3)**	**4610 (4143 to 5265)**	**14·2 (−0·3 to 47·9)**	**217 746 (192 461 to 253 543)**	**23·5 (6·2 to 58·7)**
Burundi	125 (100 to 152)	11·3 (−11·4 to 39·3)	129 (111 to 148)	1·0 (−13·4 to 18·8)	5816 (4543 to 7226)	11·4 (−13·5 to 42·3)
Comoros	11 (9 to 15)	24·5 (−4·9 to 83·3)	10 (9 to 12)	17·4 (−1·3 to 59·2)	496 (400 to 644)	20·7 (−6·5 to 73·7)
Djibouti	14 (11 to 18)	34·2 (−11·7 to 188·5)	13 (10 to 14)	25·8 (−1·0 to 141·0)	593 (441 to 760)	27·9 (−12·5 to 164·0)
Eritrea	73 (59 to 97)	36·8 (−0·2 to 114·5)	66 (56 to 79)	22·7 (−1·0 to 71·6)	3265 (2695 to 4200)	34·4 (1·9 to 104·4)
Ethiopia	1305 (1059 to 1720)	23·4 (−2·6 to 58·5)	1164 (1032 to 1437)	5·3 (−5·7 to 21·2)	53 464 (43 380 to 69 382)	17·9 (−5·6 to 45·6)
Kenya	414 (287 to 537)	35·4 (5·5 to 153·7)	491 (285 to 615)	15·1 (−0·3 to 106·8)	18 590 (12 136 to 24 765)	34·7 (6·7 to 141·3)
Madagascar	289 (232 to 352)	22·0 (−10·3 to 80·6)	309 (270 to 347)	16·3 (−1·9 to 54·2)	12 852 (10 415 to 15 830)	19·8 (−10·3 to 71·1)
Malawi	167 (132 to 207)	17·8 (−16·7 to 87·6)	162 (146 to 184)	10·6 (−7·9 to 46·3)	7315 (5873 to 9087)	10·5 (−19·6 to 60·0)
Mozambique	561 (442 to 742)	12·2 (−14·1 to 58·0)	489 (411 to 632)	9·4 (−7·8 to 42·6)	28 852 (22 684 to 36 222)	8·4 (−12·6 to 42·1)
Rwanda	153 (122 to 190)	35·6 (8·1 to 71·7)	143 (126 to 161)	6·5 (−3·5 to 19·3)	6941 (5543 to 8436)	32·7 (7·1 to 64·4)
Somalia	125 (100 to 153)	16·2 (−6·8 to 49·9)	123 (103 to 137)	11·7 (−0·6 to 34·6)	5564 (4375 to 6798)	13·9 (−7·5 to 43·9)
South Sudan	121 (65 to 174)	26·1 (−6·1 to 94·3)	139 (80 to 181)	11·6 (−0·8 to 52·4)	5346 (2882 to 7503)	22·0 (−6·9 to 75·8)
Tanzania	775 (636 to 1 015)	32·0 (−5·1 to 112·5)	662 (587 to 797)	22·0 (−1·1 to 82·1)	34 825 (28 826 to 44 349)	28·4 (−3·3 to 96·1)
Uganda	470 (361 to 588)	45·0 (−1·0 to 121·9)	473 (379 to 553)	29·8 (−1·7 to 81·0)	21 876 (17 588 to 26 779)	42·3 (3·9 to 112·3)
Zambia	264 (170 to 454)	58·7 (−8·2 to 191·6)	233 (169 to 371)	39·7 (−0·8 to 141·0)	11 948 (8066 to 19 533)	48·5 (−8·3 to 160·5)
**Central sub-Saharan Africa**	**1206 (945 to 1 428)**	**15·5 (2·7 to 29·8)**	**1384 (1177 to 1578)**	**0·3 (−13·6 to 10·7)**	**58 205 (48 384 to 69 826)**	**13·9 (−3·8 to 30·3)**
Angola	276 (205 to 364)	33·6 (0·5 to 85·1)	296 (253 to 349)	2·9 (−8·7 to 13·8)	14 106 (10 646 to 18 765)	32·4 (1·5 to 77·3)
Central African Republic	54 (42 to 66)	11·2 (−12·7 to 42·7)	67 (57 to 77)	6·8 (−2·9 to 21·2)	2467 (1889 to 3128)	13·0 (−12·9 to 43·1)
Congo (Brazzaville)	71 (48 to 120)	18·7 (−12·7 to 60·8)	75 (54 to 126)	7·6 (−6·5 to 21·6)	3232 (2096 to 5695)	20·1 (−10·8 to 60·4)
DR Congo	762 (532 to 953)	10·2 (−9·0 to 28·6)	901 (706 to 1 043)	−2·1 (−22·0 to 14·1)	36 573 (28 328 to 45 197)	7·7 (−17·2 to 29·4)
Equatorial Guinea	12 (6 to 22)	22·2 (−32·1 to 105·0)	15 (11 to 26)	17·5 (−5·6 to 59·0)	495 (268 to 930)	12·9 (−34·0 to 76·3)
Gabon	32 (21 to 55)	34·3 (−7·0 to 97·0)	30 (22 to 53)	19·1 (−1·3 to 55·2)	1331 (858 to 2412)	35·0 (−5·7 to 90·1)

DALYs=disability-adjusted life-years. UI=uncertainty interval.

Between 1990 and 2016, age-standardised incidence rates increased in all SDI quintiles (not significant in the high and low-middle SDI quintiles; table). Age-standardised death rates decreased in the high, high-middle, and middle SDI quintiles (not significant). They increased significantly in the low and low-middle SDI quintiles (table). Age-standardised DALY rates decreased in the high, high-middle, and middle SDI quintiles (not significant in the middle SDI quintile), and increased in the low and low-middle SDI quintiles (not significant in the low-middle SDI quintile (table).

Age-standardised incidence rates increased by SDI quintile with 1·98 per 100 000 person-years (95% UI 1·74–2·19) in the low SDI quintile, 2·37 per 100 000 person-years (2·16–2·69) in the low-middle, 4·63 per 100 000 person-years (4·05–4·98) in the middle, 6·36 per 100 000 person-years (5·80–6·81) in the high-middle, and 6·91 per 100 000 person-years (5·71–7·53) in the high SDI quintile. However, age-standardised death rates varied differently by SDI quintile with the highest rates observed in the high-middle (3·79 per 100 000 person-years [3·34–4·08]), followed by the high (3·64 per 100 000 person-years [2·99–3·86]), the middle (3·50 per 100 000 person-years [3·03–3·85]), the low (2·27 per 100 000 person-years [1·95–2·53]), and the low-middle SDI quintile (2·20 per 100 000 person-years [1·99–2·53]). Age-standardised DALY rates also varied by SDI quintile with the highest rates observed in the high-middle (127·28 per 100 000 person-years [113·33–136·88]), followed by the middle (116·59 per 100 000 person-years [102·24–129·29]), the high (114·37 per 100 000 person-years [97·60–126·31]), the low-middle (76·68 per 100 000 person-years [69·42–87·79]), and the low SDI quintile (74·19 per 100 000 person-years [64·27–83·77]).

**Figure 1:**
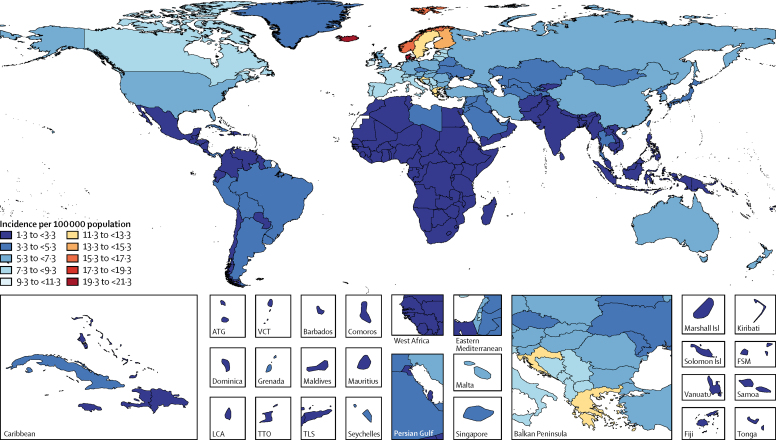
Age-standardised incidence of CNS cancer per 100 000 population for both sexes, 2016 ATG=Antigua and Barbuda. FSM=Federated States of Micronesia. Isl=Island. LCA=Saint Lucia. TLS=Timor-Leste. TTO=Trinidad and Tobago. VCT=Saint Vincent and the Grenadines.

Age-standardised incidence rates were highest in western Europe, east Asia, and central Europe and were lowest in Oceania and central and eastern sub-Saharan Africa (appendix 2). Regarding comparisons of incidence rates for specific countries, the highest age-standardised incidence rates were observed for Nordic countries (Iceland, 20·76 per 100 000 person-years [95% UI 16·18–24·66]; Denmark, 19·35 per 100 000 person-years [15·45–22·22]; Norway, 17·27 per 100 000 person-years [13·41–20·02]; Finland, 13·52 per 100 000 person-years [10·81–16·69]), and Luxembourg (16·20 per 100 000 person-years [12·52–20·88]; figure 1). In terms of absolute numbers, east Asia was the region with the most incident cases of CNS cancer for both sexes in 2016 (108 000 [98 000–122 000]), followed by western Europe (49 000 [37 000–54 000]), and south Asia (31 000 [29 000–37 000]). The top three countries with the highest number of incident cases were China, the USA, and India.

Age-standardised death rates were the highest in central Europe, tropical Latin America, and Australasia (appendix 2). The highest age-standardised death rates in specific countries were observed for Palestine (8·33 per 100 000 person-years [95% UI 7·05–9·31]), Albania (7·22 per 100 000 person-years [5·52–8·50]), Bosnia and Herzegovina (7·17 per 100 000 person-years [5·54–8·90]), and Iceland (7·10 per 100 000 person-years [5·74–8·00]). Most deaths occurred in east Asia, western Europe, and south Asia (table). The top three countries with the most deaths were China, India, and the USA.

Age-standardised DALY rates were the highest in central Europe, tropical Latin America, and eastern Europe. Most DALYs occurred in east Asia (2·0 million DALYs [95% UI 1·7–2·2]), south Asia (1·1 million [1·0–1·3]), and western Europe (722 000 [574 000–798 000]; table). The top three countries with the most DALYs were China, India, and the USA.

**Figure 2:**
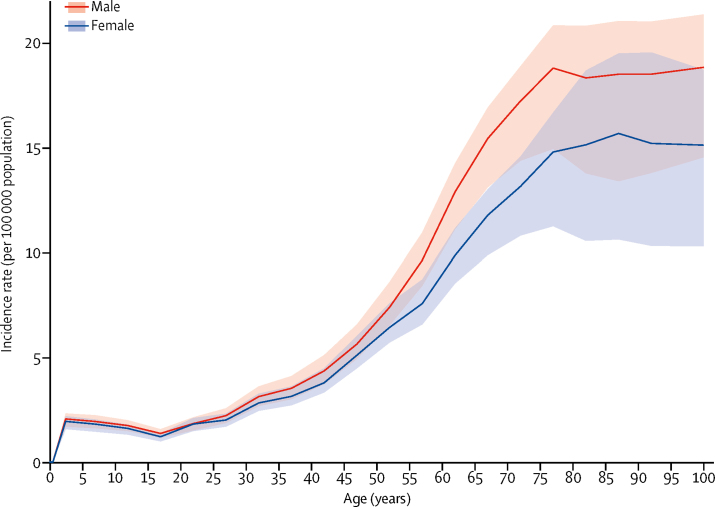
Global age-standardised incidence rate per 100 000 population of CNS cancer by age and sex, 2016 Shaded areas show 95% uncertainty intervals.

Incidence of CNS cancers had a peak in early childhood (<5 years of age) and increased after 15 years of age, with no difference in incidence rates by sex during childhood but a diverging incidence between sexes with increasing age, leading to a higher incidence in men than women, albeit this difference was not significant (figure 2).

**Figure 3:**
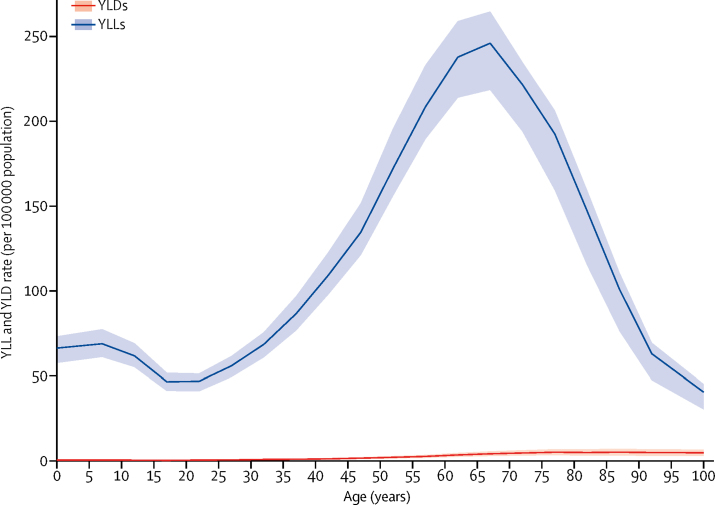
Global age-specific years lived with disability (YLDs) and years of life lost (YLLs) rates per 100 000 population due to CNS cancer, 2016 Shaded areas show 95% uncertainty intervals.

**Figure 4:**
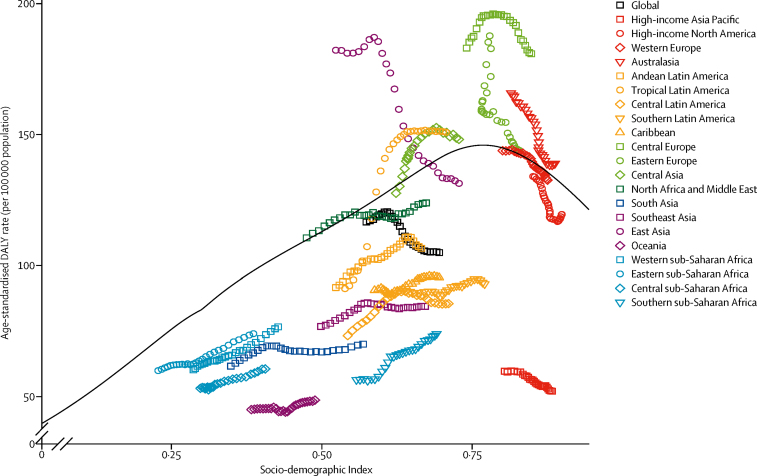
Age-standardised disability-adjusted life-year (DALY) rates per 100 000 population for CNS cancer by 21 Global Burden of Disease regions and Socio-demographic Index (SDI), 1990–2016 In all regions, SDI has increased over time so progress in SDI is associated with points further to the right and later years for a given region. The black line represents expected values based on SDI.

DALYs for CNS cancer were driven by YLLs rather than YLDs (figure 3), with YLLs peaking between 65 and 69 years of age. When analysing the pattern of DALYs by SDI, distinct patterns were observed (figure 4). Based on estimates for all countries and years, the expected pattern of age-standardised DALY rates and SDI is one of a steady increase until SDI around 0·8 and then a modest decline. However, regional patterns show large deviations from this pattern. Some regions had rising DALY rates with improvements in SDI, while others had decreasing rates or did not have a monotonic relationship with SDI. Also, among high-income countries, the high-income Asia Pacific region stood out with low DALY rates. Generally, the large regional variation around the expected pattern based on SDI suggests that factors other than sociodemographic development are responsible for most of the variation in disease burden of CNS cancer.

When analysing age-standardised mortality to incidence ratios by SDI (appendix 1), for SDI of 0·55 and greater, age-standardised mortality decreased, suggesting improved survival with higher SDI.

## Discussion

For GBD 2016, we analysed cancer registry and vital registration system data to calculate the incidence, mortality, and DALYs for primary CNS cancer. Our results show that the global burden of CNS cancer increased between 1990 and 2016, as evidenced by an increase in incident cases, deaths, and DALYs. However, despite increasing age-standardised incidence rates, age-standardised DALYs and age-standardised death rates decreased between 1990 and 2016 (albeit the change was not significant), possibly due to improved treatment and timelier, accurate diagnosis. Also, for most regions, the mortality to incidence ratio decreased with improvements in SDI and over time, which can be seen as a surrogate for improved survival. Our estimates are in line with other major efforts to quantify CNS cancer incidence and related deaths worldwide. The GLOBOCAN study estimated 256 213 incident cases and 189 382 deaths in the year 2012.^14^ GBD estimates 287 893 incident cases (95% UI 256 282–300 541) and 208 163 deaths (95% UI 188 461–219 882) for the same year. The GBD and GLOBOCAN are also consistent in showing large regional variation in the age-standardised incidence rates of CNS cancer, with the highest rates occurring in Europe and North America and the lowest rates occurring in Africa and parts of Asia.

Perhaps the most substantial global health challenge related to CNS cancer is the requirement of highly specialised medical and surgical care for diagnosis and long-term management. No simple, population-wide screening test is available for CNS cancer to allow for early, uniform detection; moreover, symptoms such as headache or seizure are often too common and non-specific to signal the need for further radiological testing. Headaches are the most common form of neurological morbidity worldwide, but few patients with headaches have CNS cancers.^26^

Patients with CNS cancer often present with a spectrum of non-specific symptoms and signs and progress to life-threatening conditions before definitive radiological diagnosis. Diagnosis and subsequent treatment planning require the use of advanced and costly imaging modalities not readily accessible in many areas. However, over the time period studied, such technologies were becoming more widely disseminated.^27^ Despite efforts in GBD to correct for underascertainment, ascertainment bias could partly explain the increased incidence of CNS cancer during this time, but the degree to which this bias contributes to overall increase in age-standardised incidence rates requires further study.

Optimal treatment paradigms for primary CNS cancer consist of multidisciplinary approaches that combine biopsy or aggressive surgical resection with postoperative radiation and chemotherapy, when appropriate.^28^ Patients require access to neurosurgical services, intensive care units, and highly specialised radiation and neuro-oncology services that are mainly located in urban areas and in countries with advanced health-care systems.^3^, ^4^ Moreover, the relative infrequency of CNS cancer compared with other cancers in adults makes them a low priority for low-resource settings. As such, the disparity in access to these services is amplified across the sociodemographic spectrum. However, our analysis shows that mortality to incidence ratios decrease with improvements in socioeconomic development, which can be interpreted as improved survival with higher SDI. This result is consistent with the improved survival for CNS cancer over time, observed by the National Cancer Institute's Surveillance, Epidemiology, and End Results programme (relative 5-year survival probability increased from 26·8% for people diagnosed in 1990 to 36·1% for those diagnosed in 2009).^29^ This result is also consistent with findings from the CONCORD-3 study,^30^ which included aggregated data from 37·5 million patients across 15 years from 322 population-based cancer registries in 71 countries. Survival for CNS cancer was stable across that time period but did improve by 3–10% in several higher SDI regions, including high-income North America (USA, Canada), western Europe (Iceland, Norway, Sweden, the UK, Denmark, France, Switzerland), and high-income Asia Pacific (South Korea, Singapore).

On a global scale, the age-standardised incidence rate of CNS cancer is increasing but DALYs are decreasing. This relationship is also true for higher SDI quintiles but is inverted for the low-middle and low SDI quintiles. These findings show that DALYs related to CNS cancer are disproportionately represented in lower SDI regions and are likely to be reflective of a lack of access to the highly specialised services needed to treat these complex diseases. These disparities are likely to result in both a delay in diagnosis and an inability to effectively implement treatment regimens that would prevent or delay mortality. The heterogeneity observed in CNS cancer incidence probably reflects a combination of multiple factors, including genetic predisposition, environmental exposures, and the above-mentioned effects of access to health care. Previous studies have suggested that CNS cancer, in particular glioma, is more common in white populations than Asian or African populations.^31^ Our data support this finding. The highest incidence rates were in western and central Europe, and the lowest rates were in Africa. Evidence suggested this pattern was independent of SDI. For example, in the highest SDI regions, the incidence of CNS cancer and associated DALYs was more than three times higher in central Europe than in high-income Asia Pacific. Broad-scale genetic susceptibilities could account for the difference in incidence across various populations, particularly when considering that regions of similar SDI should have equal access to necessary diagnostic and treatment modalities.

It is also important to note, however, that environmental factors and exposures are likely to be highly variable across these populations. A positive association with ionising radiation and negative association with atopic conditions are the only risk factors that are consistently supported by evidence.^32^, ^33^, ^34^, ^35^, ^36^ However, incidence of atopic conditions is generally higher in high SDI countries, in which we found higher CNS cancer incidence rates. Explanations for this finding are that atopic conditions might not be causal factors for considerable proportions of CNS cancer or, more probably, the association is true at the individual but not necessarily the population level. The degree to which environmental factors are responsible for regional variance in incidence requires further study. Unfortunately, detailed analysis of the relative effects of various other epidemiological risk factors in populations has not supported any causative relationships.^32^, ^33^, ^34^, ^35^, ^36^ Perhaps by identifying the large heterogeneities in incidence, the GBD study can help direct research to identify risk factors or genetic predispositions.

The largest limitation for the GBD estimates of CNS cancer is the aggregation of all malignant CNS tumours into a single group. Given the large heterogeneity in outcomes between low-grade and high-grade brain tumours, and between gliomas, tumours of the meninges, and other CNS tumour histologies, the analyses of CNS cancer as a single group should be seen as a first step until more detailed analyses can be done. With increasing availability of diagnostic tools, cancer registry data quality is improving. However, the unavailability of advanced imaging and radiologists, neurologists, oncologists, and neurosurgeons in many locations will clearly affect the diagnostic accuracy and therefore also the registry and death certificate data. Coding of CNS metastases as primary CNS tumours and inclusion of benign tumours in the malignant category are examples of data deficiencies that make it difficult to distinguish between measurement error and true variation. A strength in the GBD estimation is the use of predictive covariates in the estimation process. However, given the absence of known strong environmental and genetic risk factors for CNS cancer, only covariates that are predictive of clinical outcomes (eg, access to medical care for diagnosis and treatment) should be used rather than covariates, such as alcohol consumption, that have not been found to be linked to CNS cancer incidence or mortality.

We present a detailed account of the distribution of CNS cancer across the globe and we explore associations between incidence, DALYs, mortality to incidence ratio, and various demographic factors. The global burden of CNS cancer has increased over the past 25 years. However, the relationship between the mortality to incidence ratio and SDI suggests that access to early detection and treatment leads to improved outcomes. This analysis can be used to inform resource allocation and strategic planning on a global scale and highlights the need for further research into underlying risk factors and associations with genetic susceptibilities that could explain the large heterogeneity in CNS cancer incidence.
