# Supplementary material for: Global, regional, and national burden of brain and other CNS cancer, 1990–2016: a systematic analysis for the Global Burden of Disease Study 2016
Source: Lancet Neurol. 2019 Apr;18(4):376–93. doi: 10.1016/S1474-4422(18)30468-X (PMC6416167; doi:10.1016/S1474-4422(18)30468-X)
Supplement: Supplementary appendix 1 [file mmc1.pdf]

THE LANCET
Neurology

Supplementary appendix

This appendix formed part of the original submission and has been peer reviewed. We post it as supplied by the authors.

Supplement to: GBD 2016 Brain and Other CNS Cancer Collaborators. Global, regional, and national burden of brain and other CNS cancer, 1990–2016: a systematic analysis for the Global Burden of Disease Study 2016. *Lancet Neurol* 2019; published online Feb 20. http://dx.doi.org/10.1016/S1474-4422(18)30468-X.

Online Appendix to "The Global Burden of Brain and Nervous System Cancer, 1990 to 2016: A Systematic Analysis for the Global Burden of Disease Study"

Contents

Summary of General Global Burden of Disease Study Methods ... 3

Cancer specific methods ... 5

- Definition of indicator ... 5
- Data sources ... 5
  - Cancer incidence data sources ... 5
  - Mortality/incidence ratio data sources ... 5
  - Cancer mortality data sources ... 5
  - Bias of categories of input data ... 6
- Data analysis ... 6
  - Cancer registry data formatting ... 6
- Cause of death database formatting ... 7
- CODEm models ... 7
- CodCorrect ... 8
- Incidence estimation ... 8
- Prevalence and YLD estimation ... 8

Tables ... 9

References ... 14

eFigure 1: Flowchart GBD cancer mortality, YLL estimation ... 11
eFigure 2: Flowchart GBD cancer incidence, prevalence, YLD estimation ... 12
eFigure 3: Age-standardised mortality to incidence ratios for brain and nervous system cancer by 21 GBD regions by socio-demographic index (SDI), 1990-2016 ... 13

eTable 1: GATHER checklist of information that should be included in reports of global health estimates, with description of compliance and location of information for GBD 2016. ... 9

Summary of General Global Burden of Disease Study Methods

The Institute for Health Metrics and Evaluation with a growing collaboration of scientists produces annual updates of the Global Burden of Disease study. Estimates span the period from 1990 to the most recent completed year. By the time of the release of GBD 2016 in September 2017, there were over 2,700 collaborators in 132 countries who contributed to this global public good. Annual updates allow incorporation of new data and method improvements to ensure that the most up-to-date information is available to policy makers in a timely fashion to help make resource allocation decisions. In this analysis, we have aggregated results from GBD 2016 for 15 disease and injury outcomes that are generally cared for by neurological services. These include infectious conditions (tetanus, meningitis, encephalitis), stroke, brain and other nervous system cancers, traumatic brain injury, and spinal cord lesion which are classified outside the more narrowly defined category of neurological disorders in GBD (ie, Alzheimer's disease and other dementias, Parkinson's disease, multiple sclerosis, motor neuron disease, idiopathic epilepsy, migraine, tension-type headache, and a rest category of less common other neurological disorders). Compared to a previous analysis based on GBD 2015,[1] we were able to add the non-fatal outcomes of traumatic brain injury and spinal cord lesion, and medication overuse headache is no longer included as a separate cause but quantified as a consequence of the underlying headache types.

In the methods section of this overview paper we present a summary of the general methods of the global burden of disease. In the accompanying disease-specific papers we concentrate on methods that are specific to each disorder. The guiding principle of GBD is to assess health loss due to mortality and disability comprehensively, where we define disability as any departure from full health. In GBD 2016, estimates were made for 195 countries and territories, and 579 subnational locations, for 27 years starting from 1990, for 23 age groups and both sexes. Deaths were estimated for 264 diseases and injuries, while prevalence and incidence were estimated for 328 diseases and injuries. In order to allow meaningful comparisons between deaths and non-fatal disease outcomes as well as between diseases, the data on deaths and prevalence are summarised in a single indicator, the disability-adjusted life-year (DALY). DALYs are the sum of years of life lost (YLLs) and years lived with disability (YLDs). YLLs are estimated as the multiplication of counts of death and a standard, "ideal", remaining life expectancy at the age of death. The standard life expectancy is derived from the lowest observed mortality rates in any population in the world greater than 5 million.[2] YLDs are estimated as the product of prevalence of individual consequences of disease (or "sequelae") times a disability weight that quantifies the relative severity of a sequela as a number between zero (representing "full health") and 1 (representing death). Disability weights have been estimated in nine population surveys and an open-access internet survey in which respondents are asked to choose the "healthier"[3] between random pairs of health states that are presented with a short description of the main features.

All-cause mortality rates are estimated from vital registration data in countries with complete coverage. For other countries, the probabilities of death before age 5 and between ages 15 and 60 are estimated from censuses and surveys asking mothers to provide a history of children ever born and those still alive, and surveys asking adults about siblings who are alive or have passed away. Using model life tables, these probabilities of death are transformed into age-specific death rates by location, year, and sex. GBD has collated a large database of cause of death data from vital registrations and verbal autopsy surveys in which relatives are asked a standard set of questions to ascertain the likely cause of death, supplemented with police and mortuary data for injury deaths in countries with no other data. For countries with vital registration data, the completeness is assessed with demographic methods based on comparing recorded deaths with population counts between two successive censuses. The cause of death information is provided in a large number of different classification systems based on versions of the International Classification of Diseases or bespoke classifications in some countries. All data are mapped into the disease and injury categories of GBD. All classification systems contain codes that are less informative because they lack a specific diagnosis (eg, unspecified cancer) or refer to codes that cannot be underlying cause of death (eg, low back pain or senility) or are intermediate causes (eg, heart failure or sepsis). Such deaths are redistributed to more precise underlying causes of death.[4] After these redistributions and corrections for under-registration, the data are analysed in CODEm (cause of death ensemble model), a highly systematised tool that runs many different models on the same data and chooses an ensemble of

models that best reflects all the available input data. Models are chosen with variations in the statistical approach ("mixed effects" of spatiotemporal Gaussian Process Regression), in the unit of analysis (rates or cause fractions), and the choice of predictive covariates. The statistical performance of all models is tested by holding out 30% of the data and checking how well a model covers the data that were held out. To enforce consistency from CODEm, the sum of all cause-specific mortality rates is scaled to that of the all-cause mortality rates in each age, sex, location, and year category.

Non-fatal estimates are based on systematic reviews of published papers and unpublished documents, survey microdata, administrative records of health encounters, registries, and disease surveillance systems. Our Global Health Data Exchange (GHDx, http://ghdx.healthdata.org/) is the largest repository of health data globally. We first set a reference case definition and/or study method that best quantifies each disease or injury or consequence thereof. If there is evidence of a systematic bias in data that used different case definitions or methods compared to reference data we adjust those data points to reflect what its value would have been if measured as the reference. This is a necessary step if one wants to use all data pertaining to a particular quantity of interest rather than choosing a small subset of data of the highest quality only. DisMod-MR 2.1, a Bayesian meta-regression tool, is our main method of analyzing non-fatal data. It is designed as a geographical cascade where a first model is run on all the world's data, which produces an initial global fit and estimates coefficients for predictor variables and the adjustments for alternative study characteristics. The global fit adjusted by the values of random effects for each of seven GBD super-regions, the coefficients on sex and country predictors, are passed down as data to a model for each super-region together with the input data for that geography. The same steps are repeated going from super-region to 21 region fits and then to 195 fits by country and where applicable a further level down to subnational units. Below the global fit, all models are run separately by sex and for six time periods: 1990, 1995, 2000, 2005, 2010, and 2016. During each fit all data on prevalence, incidence, remission (ie, cure rate) and mortality are forced to be internally consistent. For most diseases, the bulk of data on prevalence or incidence is at the disease level with fewer studies providing data on the proportions of cases of disease in each of the sequelae defined for the disease. The proportions in each sequela are pooled using DisMod-MR 2.1 or meta-analysis, or derived from analyses of patient-level datasets. The multiplication of prevalent cases for each disease sequela and the appropriate disability weight produces YLD estimates that do not yet take into account comorbidity. To correct for comorbidity, these data are used in a simulation to create hypothetical individuals in each age, sex, location, and year combination who experience no, one, or multiple sequelae simultaneously. We assume that disability weights are multiplicative rather than additive as this avoids assigning a combined disability weight value in any individual to exceed 1, ie, be worse than a "year lost due to death". This comorbidity adjustment leads to an average scaling down of disease-specific YLDs ranging from about 2% in young children up to 17% in oldest ages.

All our estimates of causes of death are categorical: each death is assigned to a single underlying cause. This has the attractive property that all estimates add to 100%. For risks, we use a different, "counterfactual" approach, ie, answering the question: "what would the burden have been if the population had been exposed to a theoretical minimum level of exposure to a risk". Thus, we need to define what level of exposure to a risk factor leads to the lowest amount of disease. We then analyse data on the prevalence of exposure to a risk and derive relative risks for any risk-outcome pair for which we find sufficient evidence of a causal relationship. Prevalence of exposure is estimated in DisMod-MR 2.1, using spatiotemporal Gaussian Process Regression, or from satellite imagery in the case of ambient air pollution. Relative risk data are pooled using meta-analysis of cohort, case-control and/or intervention studies. For each risk and outcome pair, we evaluate the evidence and judge if the evidence falls into the categories of "convincing" or "probable" as defined by the World Cancer Research Fund.[5] From the prevalence and relative risk results, population attributable fractions are estimated relative to the theoretical minimum risk exposure level (TMREL). When we aggregate estimates for clusters of risks, eg, metabolic or behavioural risks, we use a multiplicative function rather than simple addition and take into account how much of each risk is mediated through another risk. For instance, some of the risk of high body mass index is directly onto stroke as an outcome but much of its impact is mediated through high blood

pressure, high cholesterol, or high fasting plasma glucose, and we would not want to double count the mediated effects when we estimate aggregates across risk factors.[6]

Uncertainty is propagated throughout all these calculations by creating 1,000 values for each prevalence, death, YLL, YLD, or DALY estimate and performing aggregations across causes and locations at the level of each of the 1,000 values for all intermediate steps in the calculation. The lower and upper bounds of the 95% uncertainty interval are the 25th and 975th values of the ordered 1,000 values. For all age-standardised rates, GBD uses a standard population calculated as the non-weighted average across all countries of the percentage of the population in each five-year age group for the years 2010 to 2035 from the United Nations Population Division's World Population Prospects (2012 revision).[7,8]

GBD uses a composite indicator or sociodemographic development, SDI, which reflects the geometric mean of normalised values of a location's income per capita, the average years of schooling in the population 15 and over, and the total fertility rate. Countries and territories are grouped into five quintiles of high, high-middle, middle, low-middle, and low SDI based on their 2016 values.[2]

Cancer specific methods (as previously published in "Global Burden of Disease Cancer Collaboration, Fitzmaurice C, Akinyemiju TF, et al. Global, Regional, and National Cancer Incidence, Mortality, Years of Life Lost, Years Lived With Disability, and Disability-Adjusted Life-Years for 29 Cancer Groups, 1990 to 2016: A Systematic Analysis for the Global Burden of Disease Study. JAMA Oncol 2018; published online June 2. DOI:10.1001/jamaoncol.2018.2706.")

Definition of indicator

The GBD cause list is organized in a hierarchy. Levels 1 and 2 represent general groupings. The broad group "neoplasms." which includes all cancer causes, is at Level 2 under the Level 1 group Non-communicable diseases. Level 3 includes 29 cancer groups. In this publication, estimates for the GBD cancer group "Brain and nervous system cancer", for both sexes, for the time from 1990 to 2016, and for the 5-year GBD age groups (0-5; 5-9; etc. until 95+) are presented for 195 countries or territories. All ICD10 codes pertaining to brain and nervous system cancer (C70-C72.9) are included in these estimates.

Data sources

Cancer incidence data sources

Cancer incidence was sought from individual cancer registries or aggregated databases of cancer registry data like "Cancer Incidence In Five Continents" (CI5),"[9–18] EUREG,[19] or NORDCAN.[20] Data were excluded if they were not representative of the coverage population (e.g., hospital-based registries), if they did not cover all malignant neoplasms as defined in ICD9 (140-208) or ICD10 (C00-C96) (e.g., specialty cancer registry), if they did not include data for both sexes and all age groups, if the data were limited to years prior to 1980, or if the source did not provide details on the population covered. Preference was given to registries with national coverage over those with only local coverage, except those from countries where the GBD study provides subnational estimates. A list of the data sources included for our estimates can be found in the online GBD citation tool, http://ghdx.healthdata.org/gbd-2016/data-input-sources.

Mortality/incidence ratio data sources

Most cancer registries only report cancer incidence. However, if a cancer registry also reported cancer mortality, mortality data were also extracted from the source to be used in the mortality to incidence estimation.

Cancer mortality data sources

A detailed description of the data sources and processing steps for the cause of death database can be found in the appendix to the GBD 2016 paper "Global, regional, and national age-sex specific mortality for 264 causes of death, 1980–2016: a systematic analysis for the Global Burden of Disease Study 2016."[21]

Bias of categories of input data

Bias of the input data included for the COD database is described elsewhere.[21] Cancer registry data can be biased in multiple ways. A high proportion of ill-defined cancer cases in the registry data requires redistribution of these cases to other cancers, which introduces a potential for bias. Changes between coding systems can lead to artificial differences in disease estimates; however, we adjust for this bias by mapping the different coding systems to the GBD causes. Underreporting of cancers that require advanced diagnostic techniques like brain and nervous system cancer can be an issue in cancer registries from low-income countries. On the other hand, misclassification of metastatic sites as primary cancer can lead to overestimation of cancer sites that are common sites for metastases like the brain. Since many cancer registries are located in urban areas, the representativeness of the registry for the general population can also be problematic. The accuracy of mortality data reported in cancer registries usually depends on the quality of the vital registration system. If the vital registration system is incomplete or of poor quality, the mortality-to-incidence ratio can be biased to lower ratios.

Data analysis

Flowcharts describing the conceptual overview of the data processing are available in eFigure 1 and eFigure 2.

Cancer registry data formatting

Cancer registry data went through multiple processing steps before integration with the COD database. First, the original data were transformed into standardized files, which included standardization of format, categorization, and registry names (#1 in eFigure 1).

Second, some cancer registries report individual codes as well as aggregated totals (e.g., C18, C19, and C20 are reported individually but the aggregated group of C18–C20 (colorectal cancer) is also reported in the registry data). The data processing step "subtotal recalculation" (#2 in flowchart) verifies these totals and subtracts the values of any individual codes from the aggregates.

In the third step (#3 in the flowchart), cancer registry incidence data and cancer registry mortality data are mapped to GBD causes.

In the fourth data processing step (#4 in the flowchart), cancer registry data were standardized to the GBD age groups. Age-specific incidence rates were generated using CI5, SEER, and NORDCAN data, while age-specific mortality rates were generated from the CoD data.[21] Age-specific weights were then generated by applying the age-specific rates to a given registry population that required age-splitting to produce the expected number of cases/deaths for that registry by age. The expected number of cases/deaths for each sex, age, and cancer were then normalized to 1, creating final, age-specific proportions. These proportions were then applied to the total number of cases/deaths by sex and cancer to get the age-specific number of cases/deaths.

In the rare case that the cancer registry only contained data for both sexes combined, the age-specific cases/deaths were split and re-assigned to separate sexes using the same weights that are used for the age-splitting process. Starting from the expected number of deaths, proportions were generated by sex for each age (e.g., if for ages 15-19 years old there are 6 expected deaths for males and 4 expected deaths for females, then 60% of the combined-sex deaths for ages 15-19 years would be assigned to males and the remaining 40% would be assigned to females).

In the fifth step (#5 in the flowchart), data for cause entries that are aggregates of GBD causes were redistributed. Examples of these aggregated causes include some registries reporting ICD10 codes C00-C14 together as, "lip, oral cavity, and pharyngeal cancer." These groups were broken down into subcauses that could be mapped to single GBD causes. In this example, those include lip and oral cavity cancer (C00-C08), nasopharyngeal cancer (C11), cancer of other parts of the pharynx (C09-C10, C12-C13), and "Malignant neoplasm of other and ill-defined sites in the lip, oral cavity, and pharynx" (C14). To redistribute the data, weights were created using the same method employed in age-sex splitting (see step four above). For the undefined code (C14 in the example) an "average all cancer" weight was used, which was generated by adding all cases rom SEER/NORDCAN/CI5 and dividing those by the combined population. Then, proportions were generated by subcause for each aggregate cause as in the sex splitting example above (see step four).

The total number of cases from the aggregated group (C00-C14) was recalculated for each subgroup and the undefined code (C14). C14 was then redistributed as a "garbage code" in step six.

In the sixth step (#6 in the flowchart), unspecified codes ("garbage code") were redistributed. Redistribution of cancer registry incidence and mortality data mirrored the process of the redistribution used in the cause of death database and has not changed compared to GBD 2013.[21]

In the seventh step (#7 in the flowchart), duplicate or redundant sources were removed from the processed cancer registry dataset. Duplicate sources were present if, for example, the cancer registry was part of the CI5 database but we also had data from the registry directly. Redundancies occurred and were removed as described in "Inclusion and Exclusion Criteria," where more detailed data were available, or when national registry data could replace regionally representative data. From here, two parallel selection processes were run to generate input data for the MI models and to generate incidence for final mortality estimation. Higher priority was given to registry data from the most standardized source when creating the final incidence input, whereas for the MI model input, only sources that reported incidence and mortality were used. This is different from GBD 2015, where mortality and incidence could come from different sources as long as they covered the same population.

In the eighth step (#8 in the flowchart), the processed incidence and mortality data from cancer registries were matched by cancer, age, sex, year, and location to generate MI ratios. These MI ratios were used as input for a three-step modeling approach using the general GBD ST-GPR approach with SDI as a covariate in the linear step mixed effects model using a logit link function. Predictions were made without the random effects. The ST-GPR model has three main hyper-parameters that control for smoothing across time, age, and geography. The time adjustment parameter (λ) was set to 2, which aims to borrow strength from neighboring time points (i.e., the exposure in this year is highly correlated with exposure in the previous year but less so further back in time). The age adjustment parameter ω was set to 0.5, which borrows strength from data in neighboring age groups. The space adjustment parameter ξ was set to 0.95 in locations with data and to 0.5 in locations without data (the higher ξ was applied when at least one age-sex group in the country of estimation had at least five unique data points. The lower ξ was applied when estimating data-scarce countries). Zeta aims to borrow strength across the hierarchy of geographical locations.[21] For the amplitude parameter in the Gaussian process regression we used 2 and for the scale we used a value of 15.

Final MI ratios were matched with the cancer registry incidence dataset in the ninth step (#9 in the flowchart) to generate mortality estimates (Incidence * Mortality/Incidence = Mortality) (#10 in the flowchart). The final mortality estimates were then uploaded into the COD database (#11 in the flowchart). Cancer-specific mortality modeling then followed the general CODEm process.[22]

Cause of death database formatting

Formatting of data sources for the cause of death database has been described in detail elsewhere (#11 in the flowchart).[21]

CODEm models

Mortality estimates for each cancer were generated using CODEm (#12 in the flowchart). Methods describing the CODEm approach have been described elsewhere.[21,22] In brief, the CODEm modeling approach is based on the principles that all types of available data should be used even if data quality varies; that individual models but also ensemble models should be tested for their predictive validity; and that the best model or sets of models should be chosen based on the out of sample predictive validity. Models were run separately for countries with extensive and complete vital registration data and countries with less VR data to prevent an inflation in the uncertainty around the estimates in "data-rich" countries. Covariates were selected based on a possible predictive relationship between the covariate and the specific cancer mortality. Level 1 covariates have a proven strong relationship with the outcome such as etiological or biological roles. Level 2 covariates have a strong relationship but not a direct biological link. Covariates that are more distal in the causal chain or are mediated through Level 1 or 2 covariates are categorized as Level 3.[22]

CodCorrect

CODEm models estimate the individual cause-level mortality without taking into account the all-cause mortality (#13 in the flowchart). To ensure that all single causes add up to the all-cause mortality and that all child-causes add up to the parent cause, an algorithm called "CodCorrect" is used (#14 and #15 in the flowchart). Details regarding the algorithm can be found elsewhere.[21]

Incidence estimation

GBD brain and nervous system cancer incidence estimates were generated by dividing final mortality estimates (after CodCorrect adjustment) by the MI ratio for brain and nervous system cancer (#1 eFigure 2). To propagate uncertainty from the MI ratios and the mortality estimates to incidence, this process was done at the 1,000-draw level. It was assumed that uncertainty in the MI ratio is independent of uncertainty in the estimated age-specific death rates.

Prevalence and YLD estimation

Prevalence is estimated as 10-year prevalence. To estimate brain and nervous system cancer prevalence, relative cancer survival was estimated by scaling cancer-specific survival between a "best case" and "worst case" survival. The methods and input data used to generate the best and worst case survival as well as to scale countries between these boundaries remained the same as for the GBD 2013 and GBD 2015 studies (# 2, 3, and 5 in the flowchart).[23] To transform relative to absolute survival (adjusting for background mortality) GBD 2016 lifetables were used (# 6 and 7 in the flowchart).[24] The access to cancer care variable to scale countries between the best and worst case survival was estimated using the following formula: (# 4 in the flowchart):

$$Access\ to\ care = 1 - \frac{Age\ standardized\ MI\ ratio_{cys} - Age\ standardized\ MI\ ratio_{min}}{Age\ standardized\ MI\ ratio_{max} - Age\ standardized\ MI\ ratio_{min}}$$

c=country; y=year; s=sex; Age-standardized MI $ratio_{min}$=lowest MI ratio for all countries and years; Age-standardized MI $ratio_{max}$=highest MI ratio for all countries and years

Duration of the disease phases were 5 months for diagnosis and primary therapy, 6.93 months[25] for the metastatic phase, and 1 months for the terminal phase. Total prevalence time was divided into phases 1, 3, and 4 for the population that died within 10 years, and the remaining prevalence was attributed to the controlled phase. For the population that survived beyond 10 years, prevalence person time was attributed to phase 1 and phase 2 (#8 in the flowchart). YLDs were calculated by multiplying each phase with the respective disability weight. To generate the total YLDs for brain and nervous system cancer the YLDs for each cancer sequela were added (step 9 in eFigure 2).

Tables

eTable 1: GATHER checklist of information that should be included in reports of global health estimates, with description of compliance and location of information for GBD 2016.

#	GATHER checklist item	Description of compliance	Reference
**Objectives and funding**			
1	Define the indicators, populations, and time periods for which estimates were made.	Narrative provided in paper and appendix describing indicators, definitions, and populations	Main text (Methods) and appendix
2	List the funding sources for the work.	Funding sources listed in paper	Summary (Funding)
**Data Inputs**			
For all data inputs from multiple sources that are synthesised as part of the study:			
3	Describe how the data were identified and how the data were accessed.	Narrative description of data seeking methods provided	Main text (Methods) and appendix
4	Specify the inclusion and exclusion criteria. Identify all ad-hoc exclusions.	Narrative about inclusion and exclusion criteria by data type provided; ad hoc exclusions in cause-specific write-ups	Main text (Methods) and appendix
5	Provide information on all included data sources and their main characteristics. For each data source used, report reference information or contact name/institution, population represented, data collection method, year(s) of data collection, sex and age range, diagnostic criteria or measurement method, and sample size, as relevant.	An interactive, online data source tool that provides metadata for data sources by component, geography, cause, risk, or impairment has been developed	Online data citation tools: http://ghdx.healthdata.org/gbd-2016
6	Identify and describe any categories of input data that have potentially important biases (e.g., based on characteristics listed in item 5).	Summary of known biases by cause included in appendix	Appendix
For data inputs that contribute to the analysis but were not synthesised as part of the study:			
7	Describe and give sources for any other data inputs.	Included in online data source tool	http://ghdx.healthdata.org/gbd-2016
For all data inputs:			
8	Provide all data inputs in a file format from which data can be efficiently extracted (e.g., a spreadsheet as opposed to a PDF), including all relevant meta-data listed in item 5. For any data inputs that cannot be shared due to ethical or legal reasons, such as third-party ownership, provide a contact name or the name of the institution that retains the right to the data.	Downloads of input data available through online tools, including data visualisation tools and data query tools; input data not available in tools will be made available upon request	Online data visualisation tools, data query tools, and the Global Health Data Exchange
**Data analysis**			
9	Provide a conceptual overview of the data analysis method. A diagram may be helpful.	Flow diagrams of the overall methodological processes, as well as cause-specific modelling processes, have been provided	Main text (Methods) and appendix

10	Provide a detailed description of all steps of the analysis, including mathematical formulae. This description should cover, as relevant, data cleaning, data pre-processing, data adjustments and weighting of data sources, and mathematical or statistical model(s).	Flow diagrams and corresponding methodological write-ups for each cause, as well as the databases and modelling processes, have been provided	Main text (Methods) and appendix
11	Describe how candidate models were evaluated and how the final model(s) were selected.	Provided in the methodological write-ups	Appendix
12	Provide the results of an evaluation of model performance, if done, as well as the results of any relevant sensitivity analysis.	Provided in the methodological write-ups	Appendix
13	Describe methods for calculating uncertainty of the estimates. State which sources of uncertainty were, and were not, accounted for in the uncertainty analysis.	Appendix	Appendix
14	State how analytic or statistical source code used to generate estimates can be accessed.	Appendix	http://ghdx.healthdata.org/gbd-2016-code
**Results and Discussion**			
15	Provide published estimates in a file format from which data can be efficiently extracted.	GBD 2016 results are available through online data visualisation tools, the Global Health Data Exchange, and the online data query tool	Main text, and online data tools (data visualisation tools, data query tools, and the Global Health Data Exchange)
16	Report a quantitative measure of the uncertainty of the estimates (e.g. uncertainty intervals).	Uncertainty intervals are provided with all results	Main text, appendix, and online data tools (data visualisation tools, data query tools, and the Global Health Data Exchange)
17	Interpret results in light of existing evidence. If updating a previous set of estimates, describe the reasons for changes in estimates.	Discussion of methodological changes between GBD rounds provided in the narrative of the manuscript and appendix	Main text (Methods and Discussion) and appendix
18	Discuss limitations of the estimates. Include a discussion of any modelling assumptions or data limitations that affect interpretation of the estimates.	Discussion of limitations provided in the narrative of the main paper, as well as in the methodological write-ups in the appendix	Main text (Limitations) and appendix

Figures

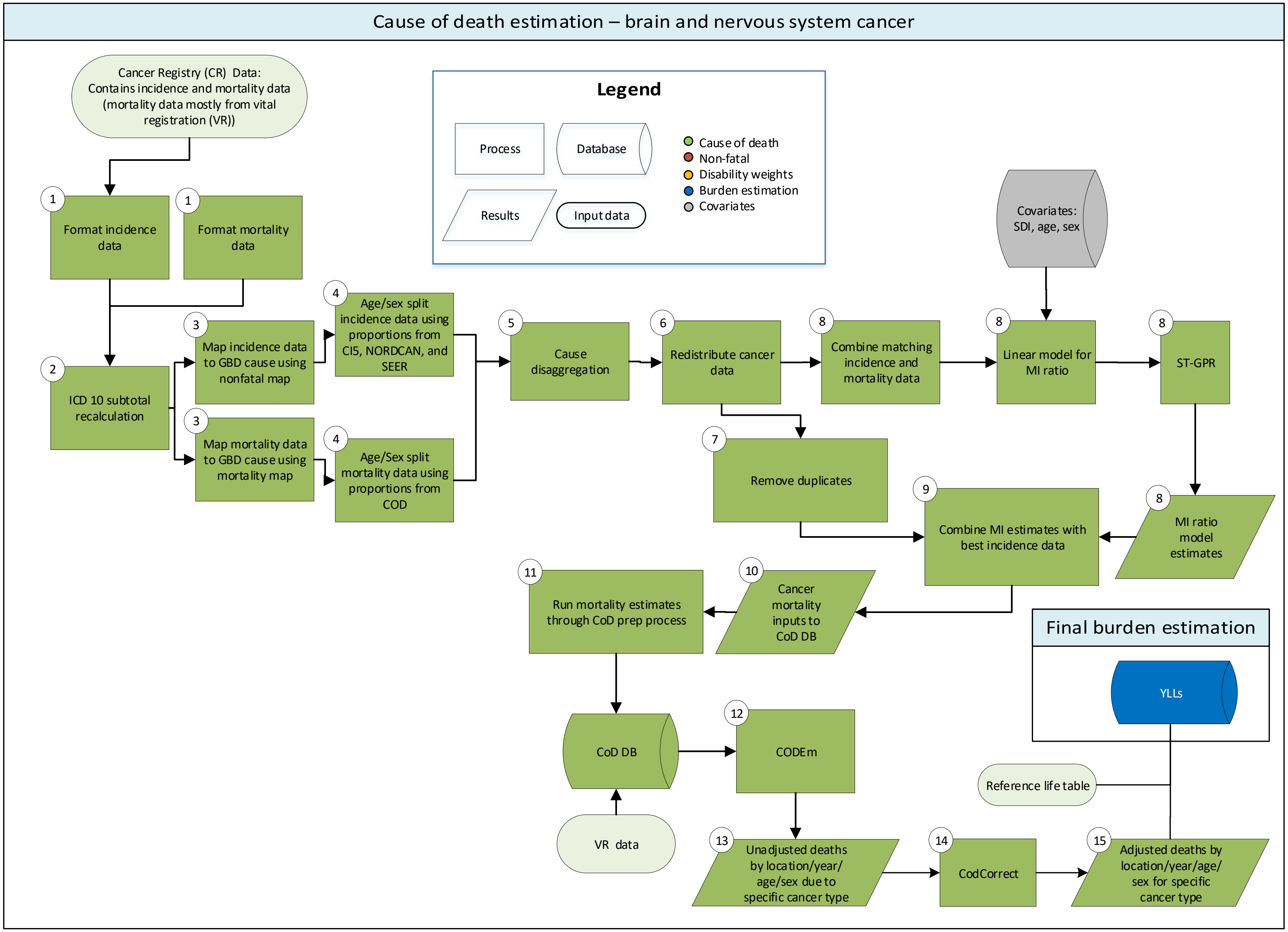

eFigure 1: Flowchart GBD cancer mortality, YLL estimation

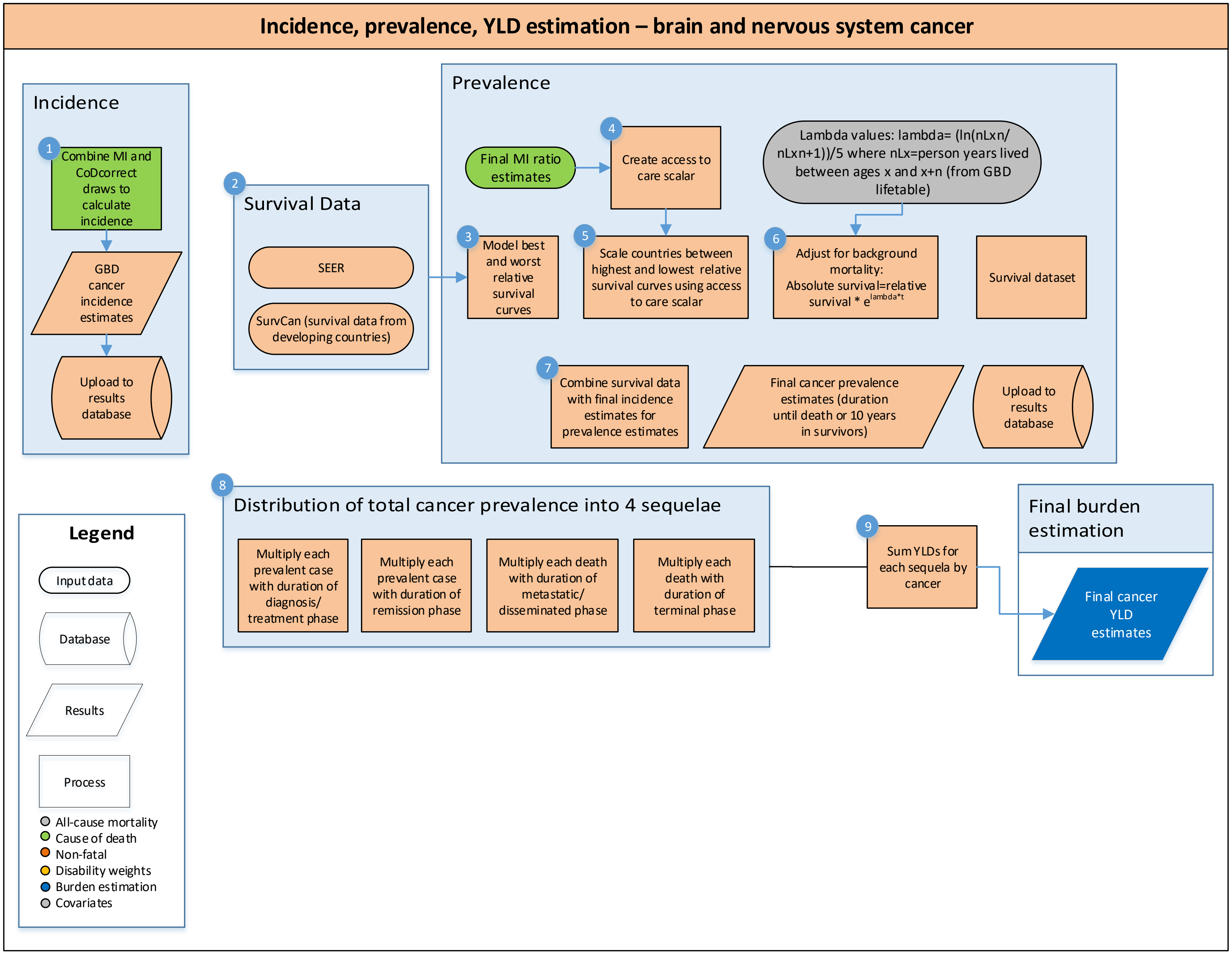

eFigure 2: Flowchart GBD cancer incidence, prevalence, YLD estimation

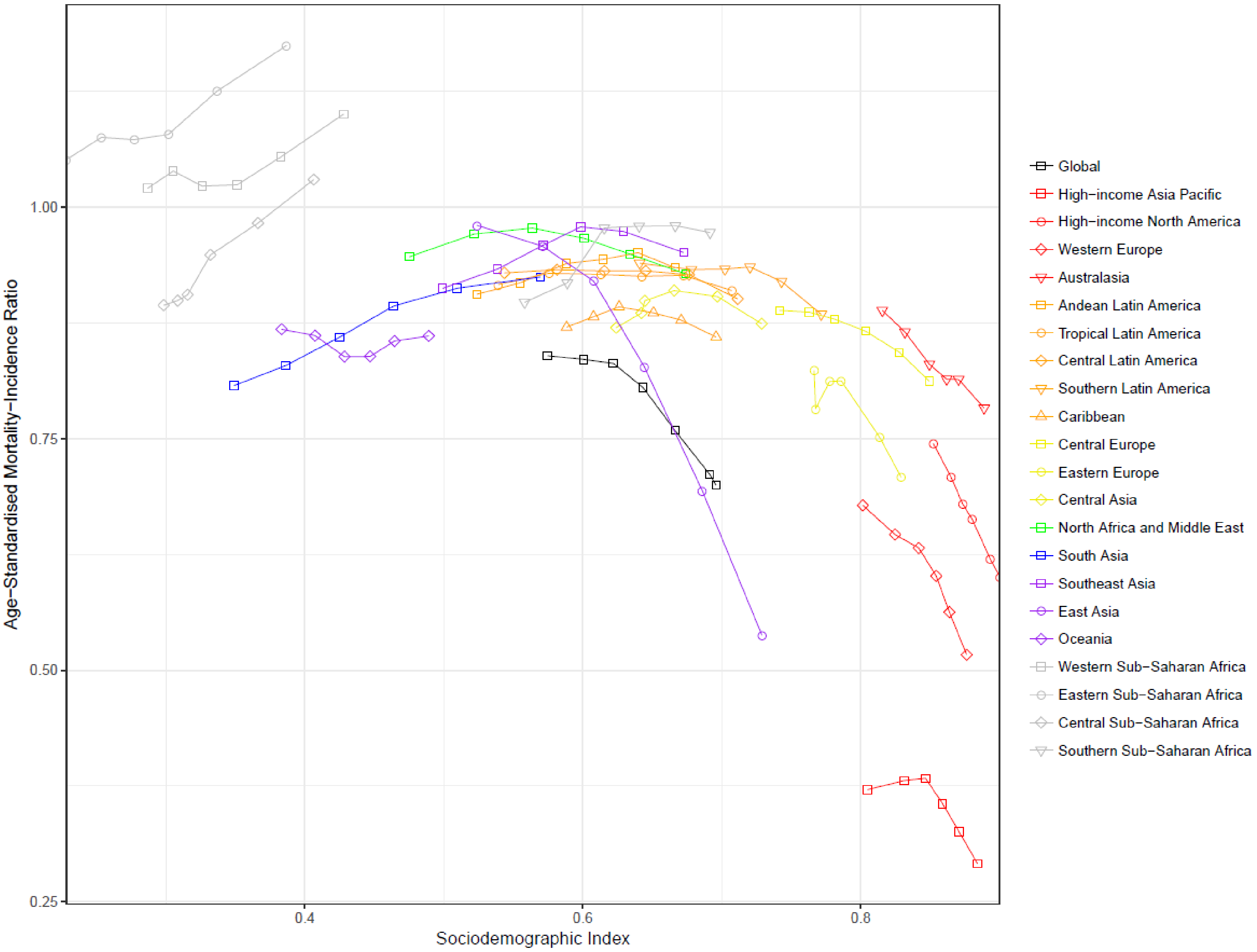

eFigure 3: Age-standardised mortality to incidence ratios for brain and nervous system cancer by 21 GBD regions by socio-demographic index (SDI), 1990-2016

References

1 GBD 2015 Neurological Disorders Collaborator Group. Global, regional, and national burden of neurological disorders during 1990-2015: a systematic analysis for the Global Burden of Disease Study 2015. *Lancet Neurol* 2017; **16**: 877–97.

2 GBD 2016 Mortality Collaborators. Global, regional, and national under-5 mortality, adult mortality, age-specific mortality, and life expectancy, 1970-2016: a systematic analysis for the Global Burden of Disease Study 2016. *Lancet Lond Engl* 2017; **390**: 1084–150.

3 Salomon JA, Haagsma JA, Davis A, *et al.* Disability weights for the Global Burden of Disease 2013 study. *Lancet Glob Health* 2015; **3**: e712-723.

4 GBD 2016 Causes of Death Collaborators. Global, regional, and national age-sex specific mortality for 264 causes of death, 1980-2016: a systematic analysis for the Global Burden of Disease Study 2016. *Lancet Lond Engl* 2017; **390**: 1151–210.

5 American Institute for Cancer Research. Food, nutrition, physical activity, and the prevention of cancer: a global perspective. Washington, DC: American Institute for Cancer Research, 2007.

6 GBD 2016 Risk Factors Collaborators. Global, regional, and national comparative risk assessment of 84 behavioural, environmental and occupational, and metabolic risks or clusters of risks, 1990-2016: a systematic analysis for the Global Burden of Disease Study 2016. *Lancet Lond Engl* 2017; **390**: 1345–422.

7 GBD 2013 Mortality and Causes of Death Collaborators. Global, regional, and national age-sex specific all-cause and cause-specific mortality for 240 causes of death, 1990-2013: a systematic analysis for the Global Burden of Disease Study 2013. *Lancet Lond Engl* 2015; **385**: 117–71.

8 United Nations Department of Economics and Social Affairs Population Division. World Population Prospects: The 2012 Revision. http://esa.un.org/unpd/wpp/Documentation/publications.htm (accessed Nov 4, 2014).

9 Doll R, Payne P, Waterhouse J. Cancer Incidence in Five Continents I. Geneva: Union Internationale Contre le Cancer, 1966.

10 Doll R, Muir C, Waterhouse J. Cancer Incidence in Five Continents II. Geneva: Union Internationale Contre le Cancer, Geneva, 1970.

11 Waterhouse J, Muir C, Correa P, Powell J. Cancer Incidence in Five Continents III. Lyon: IARC, 1976.

12 Waterhouse J, Muir C, Shanmugaratnam K, Powell J. Cancer Incidence in Five Continents IV. Lyon: IARC, 1982.

13 Muir C, Mack T, Powell J, Whelan S. Cancer Incidence in Five Continents V. Lyon: IARC, 1987.

14 Parkin D, Muir C, Whelan S, Gao Y, Ferlay J, Powell J. Cancer Incidence in Five Continents VI. Lyon: IARC, 1992.

15 Parkin D, Whelan S, Ferlay J, Raymond L, Young J. Cancer Incidence in Five Continents VII. Lyon: IARC, 1997.

16 Parkin D, Whelan S, Ferlay J, Teppo L, Thomas D. Cancer Incidence in Five Continents VIII. Lyon: IARC, 2002.

17 Curado M, Edwards B, Shin H, *et al.* Cancer Incidence in Five Continents IX. Lyon: IARC, 2007 http://www.iarc.fr/en/publications/pdfs-online/epi/sp160/CI5vol9-A.pdf.

18 Forman D, Bray F, Brewster D, *et al.* Cancer Incidence in Five Continents X. 2013. http://ci5.iarc.fr.

19 Steliarova-Foucher E, O'Callaghan M, Ferlay J, Masuyer E, Forman D, Comber H, Bray F. European Cancer Observatory: Cancer Incidence, Mortality, Prevalence and Survival in Europe. International Agency for Research on Cancer. http://eco.iarc.fr (accessed Aug 10, 2016).

20 Engholm G, Ferlay J, Christensen N, *et al.* NORDCAN--a Nordic tool for cancer information, planning, quality control and research. *Acta Oncol* 2010; **49**: 725–36.

21 Abajobir AA, Abbafati C, Abbas KM, *et al.* Global, regional, and national age-sex specific mortality for 264 causes of death, 1980–2016: a systematic analysis for the Global Burden of Disease Study 2016. *The Lancet* 2017; **390**: 1151–210.

22 Foreman KJ, Lozano R, Lopez AD, Murray CJ. Modeling causes of death: an integrated approach using CODEm. *Popul Health Metr* 2012; **10**: 1.

23 Fitzmaurice C, Dicker D, Pain A, *et al.* The Global Burden of Cancer 2013. *JAMA Oncol* 2015; published online May 28. DOI:10.1001/jamaoncol.2015.0735.

24 GBD Mortality and Causes of Death Collaborators. Global, regional, and national life expectancy, all-cause and cause specific mortality for 249 causes of death, 1980–2015: a systematic analysis for the Global Burden of Disease Study 2015. *The Lancet* 2016; published online Aug.

25 Surveillance, Epidemiology, and End Results (SEER) Program (www.seer.cancer.gov) SEER*Stat Database: Incidence - SEER 18 Regs Research Data + Hurricane Katrina Impacted Louisiana Cases, Nov 2012 Sub (1973-2010 varying) - Linked To County Attributes - Total U.S., 1969-2011 Counties, National Cancer Institute, DCCPS, Surveillance Research Program, Surveillance Systems Branch, released April 2013, based on the November 2012 submission. .
